# Plasma lipidomic profiling reveals metabolic adaptations to pregnancy and signatures of cardiometabolic risk: a preconception and longitudinal cohort study

**DOI:** 10.1186/s12916-023-02740-x

**Published:** 2023-02-13

**Authors:** Li Chen, Sartaj Ahmad Mir, Anne K. Bendt, Esther W. L. Chua, Kothandaraman Narasimhan, Karen Mei-Ling Tan, See Ling Loy, Kok Hian Tan, Lynette P. Shek, Jerry Chan, Fabian Yap, Michael J. Meaney, Shiao-Yng Chan, Yap Seng Chong, Peter D. Gluckman, Johan G. Eriksson, Neerja Karnani, Markus R. Wenk

**Affiliations:** 1 Singapore Institute for Clinical Sciences, A*STAR, Singapore, Singapore; 2 Singapore Lipidomics Incubator, Life Sciences Institute, National University of Singapore, Singapore, Singapore; 3 Department of Biochemistry, Yong Loo Lin School of Medicine , National University of Singapore, Singapore, Singapore; 4 KK Women’s and Children’s Hospital, Singapore, Singapore; 5 Duke-NUS Medical School, Singapore, Singapore; 6 Department of Pediatrics, Yong Loo Lin School of Medicine, National University of Singapore, Singapore, Singapore; 7 Lee Kong Chian School of Medicine, Nanyang Technological University, Singapore, Singapore; 8 Department of Obstetrics and Gynaecology and Human Potential Translational Research Programme, Yong Loo Lin School of Medicine, National University of Singapore, Singapore, Singapore; 9 Sackler Program for Epigenetics & Psychobiology at McGill University, Montréal, Canada; 10 Ludmer Centre for Neuroinformatics and Mental Health, Douglas Mental Health University Institute, McGill University, Montréal, Canada; 11 Centre for Human Evolution, Adaptation and Disease, Liggins Institute, University of Auckland, Auckland, New Zealand; 12 Folkhalsan Research Center, Helsinki, Finland; 13 Department of General Practice and Primary Health Care, University of Helsinki, Helsinki, Finland; 14 Bioniformatics Institute, A*STAR, Singapore, Singapore

**Keywords:** Lipidomics, Preconception, Pregnancy, Postpartum, Weight changes, Metabolic adaptations, Insulin resistance, Glucose homeostasis, Cardiometabolic risk

## Abstract

**Background:**

Adaptations in lipid metabolism are essential to meet the physiological demands of pregnancy and any aberration may result in adverse outcomes for both mother and offspring. However, there is a lack of population-level studies to define the longitudinal changes of maternal circulating lipids from preconception to postpartum in relation to cardiometabolic risk factors.

**Methods:**

LC-MS/MS-based quantification of 689 lipid species was performed on 1595 plasma samples collected at three time points in a preconception and longitudinal cohort, Singapore PREconception Study of long-Term maternal and child Outcomes (S-PRESTO). We mapped maternal plasma lipidomic profiles at preconception (*N* = 976), 26–28 weeks’ pregnancy (*N* = 337) and 3 months postpartum (*N* = 282) to study longitudinal lipid changes and their associations with cardiometabolic risk factors including pre-pregnancy body mass index, body weight changes and glycaemic traits.

**Results:**

Around 56% of the lipids increased and 24% decreased in concentration in pregnancy before returning to the preconception concentration at postpartum, whereas around 11% of the lipids went through significant changes in pregnancy and their concentrations did not revert to the preconception concentrations. We observed a significant association of body weight changes with lipid changes across different physiological states, and lower circulating concentrations of phospholipids and sphingomyelins in pregnant mothers with higher pre-pregnancy BMI. Fasting plasma glucose and glycated haemoglobin (HbA1c) concentrations were lower whereas the homeostatic model assessment of insulin resistance (HOMA-IR), 2-h post-load glucose and fasting insulin concentrations were higher in pregnancy as compared to both preconception and postpartum. Association studies of lipidomic profiles with these glycaemic traits revealed their respective lipid signatures at three physiological states. Assessment of glycaemic traits in relation to the circulating lipids at preconception with a large sample size (*n* = 936) provided an integrated view of the effects of hyperglycaemia on plasma lipidomic profiles. We observed a distinct relationship of lipidomic profiles with different measures, with the highest percentage of significant lipids associated with HOMA-IR (58.9%), followed by fasting insulin concentration (56.9%), 2-h post-load glucose concentration (41.8%), HbA1c (36.7%), impaired glucose tolerance status (31.6%) and fasting glucose concentration (30.8%).

**Conclusions:**

We describe the longitudinal landscape of maternal circulating lipids from preconception to postpartum, and a comprehensive view of trends and magnitude of pregnancy-induced changes in lipidomic profiles. We identified lipid signatures linked with cardiometabolic risk traits with potential implications both in pregnancy and postpartum life. Our findings provide insights into the metabolic adaptations and potential biomarkers of modifiable risk factors in childbearing women that may help in better assessment of cardiometabolic health, and early intervention at the preconception period.

**Trial registration:**

ClinicalTrials.gov, NCT03531658.

**Supplementary Information:**

The online version contains supplementary material available at 10.1186/s12916-023-02740-x.

## Background

Physiological changes in circulating lipid concentrations with the progression of pregnancy are necessary to support the growing foetus as well as the maternal metabolism [1, 2]. However, dysregulation of lipid metabolism is associated with adverse outcomes for both mother and child [3–5]. There is also mounting evidence suggesting that pre-pregnancy cardiometabolic health is a strong determinant of pregnancy-related outcomes [6, 7] as well as postpartum health in women. Cardiometabolic conditions are tightly associated with dysregulation of lipid metabolism [8–10]. Changes in lipid profile such as an increase in total cholesterol, triglycerides and low-density lipoprotein cholesterol (LDL-C) and decreased concentration of high-density lipoprotein cholesterol (HDL-C) are used as measures of metabolic health status and disease risk [11, 12]. However, these lipid measures do not adequately explain the complex pathophysiological underpinnings of metabolic diseases. With the advances in high throughput mass spectrometry-based lipidomics methods, the diverse and dynamic nature of the circulating lipid species in relation to anthropometric and cardiometabolic risk factors has been studied in non-pregnant populations [13, 14]. Recent studies have also highlighted the changes in metabolic profiles in pregnancy [1, 2, 15, 16]. However, there is a gap in understanding and establishing the relation of pre-pregnancy lipidomic profiles to pregnancy and postpartum states in a longitudinal manner. Gestational weight gain (GWG) and postpartum weight retention (PWR) have been associated with both maternal and child outcomes [17–21]. Excess GWG also represents a significant risk for mothers in later life [22, 23]. There is currently a lack of proper understanding of the effects of excessive GWG as well as PWR on circulating lipidomic profiles at a population-level that may offer suitable avenues for assessment and prediction of future health outcomes. Progression of pregnancy is also associated with the development of insulin resistance and disruptions in glucose homeostasis that may result in gestational diabetes with predisposition to the postpartum development of type 2 diabetes [24]. Recent studies have described the longitudinal changes in plasma lipids in normal and gestational diabetes-complicated pregnancies in both humans and animal models [25, 26]. These studies are often of a cross-sectional nature and may be limited in their interpretation of the specific cohorts and animal models. There is a lack of such human studies at population-level in multi-ethnic cohorts particularly those from Asia. Further, a better understanding of the changes in glucose metabolism from preconception to postpartum in relation to the changes in body weight and plasma lipidomic profiles will help in the identification of the potential biomarkers associated with metabolic disease risk and enhance our understanding of the pathophysiology of cardiometabolic diseases.

Here we carried out the analysis of plasma lipidomic profiles in a preconception and longitudinal cohort, Singapore PREconception Study of long-Term maternal and child Outcomes (S-PRESTO) [27]. We studied the plasma lipidomic changes from preconception into pregnancy and postpartum, and the associations of lipidomic changes with body weight changes. We also examined the associations of pre-pregnancy body mass index (BMI), measures of glucose homeostasis and insulin resistance with lipidomic data across three physiological states in order to identify their influences on circulating lipid concentrations in a longitudinal manner. This study provides a suitable framework for understanding the plasma lipidomic changes in relation to cardiometabolic health in childbearing women. At the same time, detailed investigations into the plasma lipid metabolism will advance our understanding of the role of lipids in developmental origins of the disease.

## Methods

### Study population

S-PRESTO is a preconception, longitudinal cohort study that aims to examine the impact of women’s preconception health, nutritional status and maternal mood on their up-coming pregnancy and offspring health outcomes (ClinicalTrials.gov identifier: NCT03531658) [27]. Between February 2015 and October 2017, the S-PRESTO study recruited 1032 Chinese, Malay or Indian (or any combinations thereof) women aged 18–45 years and who intended to get pregnant and deliver in Singapore. The participants were followed up for 3 visits during the preconception phase and censored at 12 months of follow-up if pregnancy was not achieved. A total of 373 women gave live birth to singletons in this study. The SingHealth Centralized Institutional Review Board granted ethical approval (reference 2014/692/D), and written informed consent was obtained from all women.

### Clinical characteristics of participants

At enrolment, interviewer-administered questionnaires were used to collect information on age, ethnicity and educational attainment (below university and university and above). Parity (nulliparous and parous) was asked at recruitment and confirmed by medical records at delivery. Breastfeeding information was collected using interviewer-administered questionnaires at month 3 postpartum. Weights and heights were measured at preconception (at recruitment), pregnancy (26–28 weeks of gestation) and postpartum (month 3). Pre-pregnancy BMI was calculated as weight at recruitment divided by height in metres squared. Gestational weight gain was calculated by subtracting weight at preconception from weight measured at pregnancy. Weight loss was calculated by subtracting weight at postpartum from weight measured at pregnancy, and postpartum weight retention was calculated by subtracting weight at preconception from weight measured at postpartum.

The participants underwent an oral glucose tolerance test (OGTT) at recruitment, 26–28 weeks of gestation and 3 months postpartum. Fasting plasma glucose concentrations (FPG) and insulin concentration were measured after an overnight fasting (8–14 h), and 2-h post-load glucose concentrations (2hPG) were measured at 2 h after taking 75 g of glucose. Gestational diabetes mellitus (GDM) status at pregnancy was diagnosed on the basis of World Health Organization (WHO) 1999 criteria: ≥7.0 mmol/L for FPG and/or ≥7.8 mmol/L for 2hPG [28]. At preconception and postpartum, type 2 diabetes (T2D, FPG ≥ 7.0 mmol/L and/or 2hPG ≥11.1 mmol/L), impaired fasting glycaemia (IFG, 6.1 mmol/L ≤ FPG < 7.0 mmol/L and 2hPG < 7.8 mmol/L) and impaired glucose tolerance (IGT, FPG < 7.0 mmol/L and 7.8 mmol/L≤ 2hPG < 11.1 mmol/L) were diagnosed based on WHO 2006 criteria [29]. HOMA-IR was calculated by the mathematical equation (insulin (mU/mL) × FPG (mmol/L)/22.5) from Matthew [30]. Plasma glucose and HbA1c concentrations were measured using the ARCHITECT c8000 Clinical Chemistry Analyser (Abbott Laboratories). Fasting insulin concentration was measured using immunoassay on Beckman DxI 800. Serum triglyceride, total cholesterol and HDL-cholesterol were measured using enzymatic colorimetric methods (Beckman AU5800 analyser, Beckman Coulter) at the National University Hospital (NUH) clinical laboratory, which was accredited by the College of American Pathologists [31]. LDL-cholesterol was calculated using the Friedewald equation (LDL-cholesterol (mmol/L) = Total cholesterol (mmol/L) − HDL-cholesterol (mmol/L) − triglyceride (mmol/L)/2.2) [32].

### Sample selection, preparation and experimental design

Based on the availability and quality of plasma samples, a total of 1600 maternal samples from S-PRESTO cohort were selected for this lipidomics study. These consisted of 978 samples at recruitment, 338 samples collected at 26–28 weeks of gestation and 284 samples collected at 3 months postpartum. A stratified randomization strategy was used to allocate the samples into five batches. Paired samples were measured in the same batch. QCs and blanks were processed and analysed along with the study samples within each batch.

### Lipid extraction and LC-MS/MS analysis

Lipid extraction was carried out according to the stratified randomization template and 390 samples (study samples as well as QCs) were extracted in one batch with a total of five batches for the current study. Lipid extraction was carried out using butanol: methanol (extraction solvent) in a ratio of 1:1 (v/v) containing 10 mM ammonium formate and class-specific internal standards as described previously [33]. Briefly, 100 μL of extraction solvent was added to each sample, vortexed for 10 s followed by water bath sonication for 60 min with temperature maintained at 18–22 °C. The samples were centrifuged at 13,000×g for 10 min. The supernatant (total lipid extract) was collected in mass spectrometry-compatible vials and stored at −80 °C for LC-MS/MS. These lipid extracts were analysed by using Agilent 6495 QQQ mass spectrometer interfaced with an Agilent 1290 series HPLC system. Lipids were separated on a ZORBAX RRHD UHPLC C_18_ column (2.1×100mm 1.8mm, Agilent Technologies) with temperature maintained at 45 °C. Mass spectrometry analysis was performed in ESI positive ion mode with dynamic multiple reaction monitoring (dMRM). Mass spectrometry settings and MRM transitions for each lipid class, subclass and individual species were kept as described previously [33]. QC samples were analysed along with the samples to monitor sample extraction efficiency as well as LC-MS performance and were subsequently used to do batch corrections.

### Data processing

Peak integration was carried out in MassHunter Quantitative software (Agilent Technologies) to select area of each individual lipid species. Manual inspection was carried out to ensure that correct peaks were picked at specific retention time. Peak areas along with retention times were exported as .csv for further analysis. Peak areas of lipid species were normalized to their class-specific internal standard as described previously for quantification [15]. Batch QCs were used to correct signal drifts across the batches based on LOESS regression method (locally polynomial regression fitting, span=0.75) [34]. Following that, lipid species were dropped if quality control coefficient of variation were greater than 25%. Finally, a total of 689 lipid species representing 36 lipid classes were used for the downstream data analysis. In addition, we removed 5 outlier samples based on principal component analysis (PCA) that resulted in 1,595 samples for further downstream analysis.

### Statistical analysis

All lipidomics data were log_10_ transformed for the downstream analyses. The unsupervised principal component analysis of lipidomics data from all the samples (*N *= 1595) was applied to identify underlying differences between three time points (preconception (*N *= 976), pregnancy (*N *= 337), postpartum (*N *= 282)). The overall analysis framework is summarized in Additional file 1: Fig. S1 and each analysis is elaborated below. There are three analyses for understanding adaptions in lipid metabolism to pregnancy and four association analyses for identifying lipid signatures of cardiometabolic risk traits in this study.

The lipidomic profiles of non-pregnant (*n *= 494, not pregnant within 12 months of recruitment) and pre-pregnant (*n *= 360, live birth) subjects at preconception were compared by linear regression after the adjustment for age, ethnicity, educational attainment, parity and pre-pregnancy BMI. The subjects with missing values of required variables or the status of dropout or pregnancy problems were excluded from this analysis. The regression coefficients (β) were converted to % change in lipid concentration between two groups. The adjusted *p*-values (*P*_adj_) were calculated by the Benjamini-Hochberg (BH) method for multiple testing correction.

The trio subjects with available lipidomic profiles at three time points (*n *= 263) were used for longitudinal comparative studies. Paired *t*-test was applied for comparative lipidomics studies between pregnancy vs. preconception, pregnancy vs. postpartum and postpartum vs. preconception. No type 2 diabetes patients were found in the trio subjects. The effect size was converted to log_2_ fold change (log_2_FC). The lipid species with the adjusted *p*-value (*P*_adj_) < 0.05 were considered to be significant.

The patterns of lipid changes across three time points were investigated based on the trends of effect sizes and the significance of adjusted *p*-values in the results of two comparative studies (pregnancy vs. preconception and pregnancy vs. postpartum). The minor differences in trends between preconception and postpartum were not considered for this pattern analysis. From preconception to pregnancy or pregnancy to postpartum, there were three possible trends, i.e. increasing, decreasing and no significant change. Therefore, the total number of patterns from preconception to postpartum was nine. Each lipid species was classified into these nine patterns.

The association between lipid change and body weight change was investigated by linear regression analysis after the adjustment for age, ethnicity, educational attainment, parity and pre-pregnancy BMI using trio subjects. Breastfeeding is not considered a confounder as it is not associated with postpartum weight retention. Three linear regression models were studied for each lipid species. Sample size in each model was slightly different due to the missing data of the required variables. First, log_2_FC of the lipid level of pregnancy to that of preconception was regressed against gestational weight gain (*n* = 261). Second, log_2_FC of the lipid level of pregnancy to that of postpartum was regressed against weight loss (*n* = 253). Last, log_2_FC of the lipid level of postpartum to that of preconception was regressed against postpartum weight retention (*n* = 253). Standardized effect size (SD/SD) was used in this study. The lipid species with the adjusted *p*-value (*P*_adj_) < 0.05 were considered to be significant. The association between changes in body weight and lipid profile (total cholesterol, HDL-cholesterol, LDL-cholesterol and triglyceride) was also examined.

The lipids associated with pre-pregnancy BMI at three time points were examined by linear regression analysis using trio subjects (*n* = 252). At preconception, pre-pregnancy BMI was studied after accounting for the effects of age, ethnicity, educational attainment and parity. At pregnancy, pre-pregnancy BMI was investigated after the adjustment of gestational weight gain, age, ethnicity, educational attainment and parity. At postpartum, pre-pregnancy BMI was analysed after the adjustment of postpartum weight retention, age, ethnicity, educational attainment and parity. Breastfeeding is not adjusted in the postpartum model as it is not associated with pre-pregnancy BMI. The regression coefficients (*β*) were converted to % change in lipid concentration per unit BMI (% change = (10^β^− 1) × 100). The overlapping lipid signatures at three time points were investigated. The lipid species with the adjusted *p*-value (*P*_adj_) < 0.05 were considered to be significant.

Association of measures of glucose homeostasis and insulin resistance with plasma lipidomic profiles at three time points was conducted by linear regression analysis using trio subjects. The adjusted confounders were slightly different in the regression models at three time points. At preconception, the adjusted confounders were pre-pregnancy BMI, age, ethnicity, education attainment and parity. At pregnancy, gestational weight gain was added in the basis of confounders at preconception. At postpartum, postpartum weight retention was added in the basis of confounders at preconception. Breastfeeding is not considered a confounder in the postpartum model as it is not associated with all the measures after the adjustment of other confounders. Sample size in each model was slightly different due to the missing data of the required variables. FPG (*n* = 249), 2hPG (*n* = 226), GDM status (57 GDM vs. 187 Normal), fasting insulin (*n* = 243) concentrations, HOMA-IR (*n* = 240) and HbA1c (*n* = 249) were regressed at three time points using trio subjects.

As the lipidomic data was with the largest sample size at preconception, comparative assessment of association studies of measures of glucose homeostasis and insulin resistance (FPG, 2hPG, IGT status, fasting insulin, HOMA-IR and HbA1c) was investigated by linear regression analysis after the adjustment for age, ethnicity, educational attainment, parity and pre-pregnancy BMI. The subjects with type 2 diabetes (*n* = 16), extremely high fasting insulin concentration (>40 mU/mL, *n* = 3) and missing values were excluded from this analysis. Finally, 936 subjects were investigated at preconception. In these association studies, effect size was calculated as % change in lipid concentration per unit trait for continuous variables, while effect size was reported as % change in lipid concentration between groups for categorical variables. Fisher’s method was applied to combine probabilities from each study into one test statistic ${X}^2=-2\sum_{i=1}^k{\mathit{\log}}_{10}\left({P}_i\right)$ (k studies). The lipid species with the adjusted *p*-value (*P*_adj_) < 0.05 were considered to be significant. All the analyses were implemented in MATLAB R2020a.

## Results

### Overview of plasma lipidomics

**Fig. 1:**
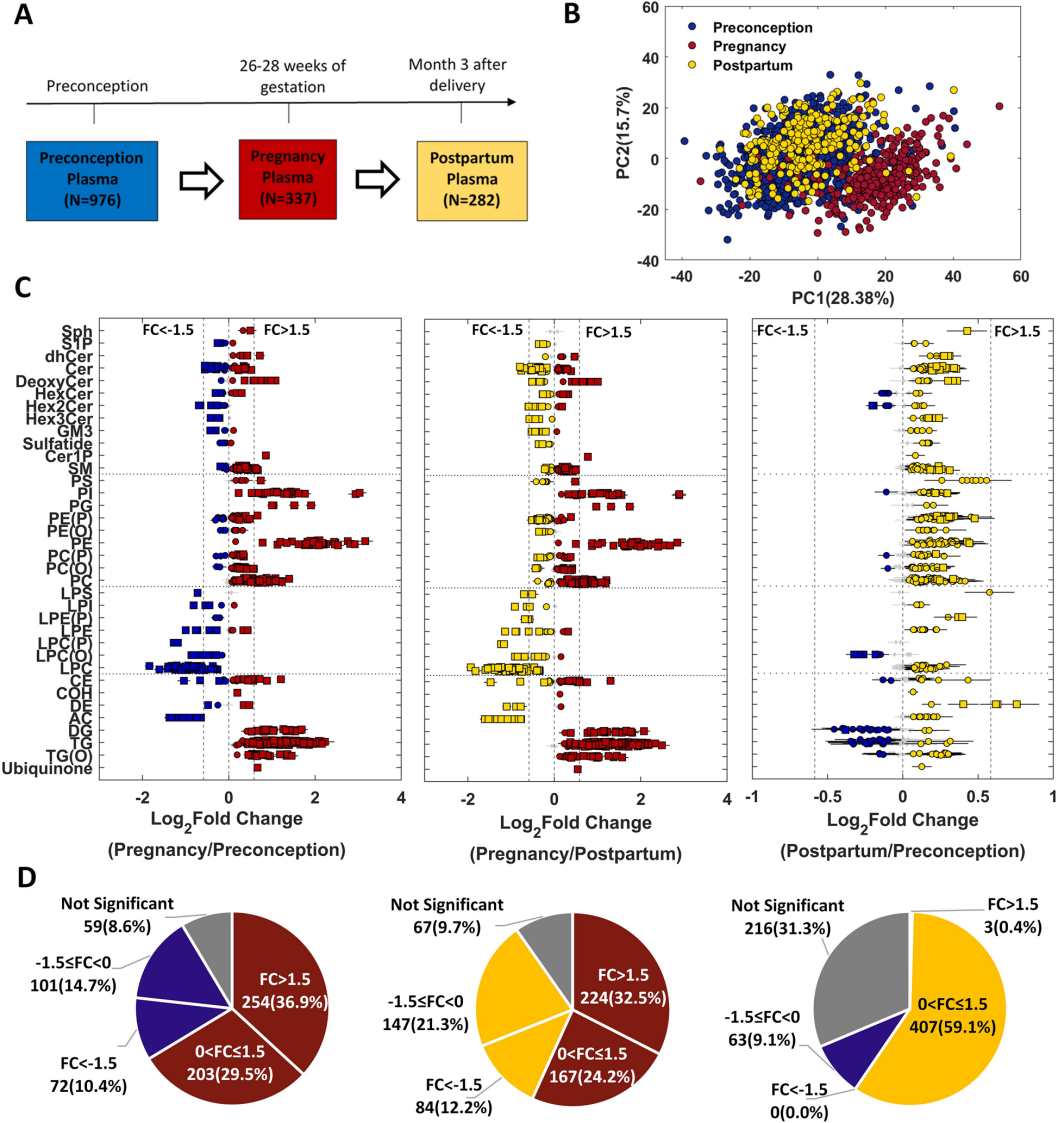
Pregnancy-induced changes in plasma lipidomic profiles. **A** Sample size and collection time points for maternal plasma samples. **B** PCA plot of lipidomics data (*N* = 1595). **C** Forest plots of three comparative studies using 263 trio subjects (pregnancy vs. preconception, pregnancy vs. postpartum and postpartum vs. preconception). Effect size—log_2_ of fold change (FC) and error bar—95% confidence interval. Diamond—*P*_adj_ ≥ 0.05 (grey), circle—*P*_adj_ < 0.05 and square—*P*_adj_ < 1.00E−10. The full names of lipid classes refer to Fig. 2C. **D** Pie charts of percentages of significant lipids in three comparative studies. Colour codes in (**D**) are consistent with (**C**)

The timeline and sample size at each time point (preconception: *N *= 976; pregnancy: *N *= 337 and postpartum: *N *= 282) are illustrated in Fig. 1A. Demographic, anthropometric and clinical characteristics of the study cohort are provided in Additional file 2: Table S1A. The principal component analysis (PCA) plot based on the lipidomic profiles (689 lipid species) of a total of 1595 participants is provided in Fig. 1B, with the first explaining 28.38% variance, and the second explaining 15.70% of the variance in the study population. The PCA plot showed distinct clusters of participants between pregnant and non-pregnant states (preconception and postpartum), and the overlap in the clusters of participants at preconception and postpartum indicated the similarities of their lipidomic profiles. Characteristics of trio participants (*n *= 263), i.e. those women with available lipidomic data across three time points, are provided in Table 1, and the comparison results of characteristics are provided in Additional file 2: Table S1B. The average time to pregnancy is around 145 days (Std = 112 days). About 62% of the participants were nulliparous with an average age of 30 years. The majority of mothers (~87%) breastfed at 3 months postpartum. As the association of breastfeeding (yes or no) with pre-pregnancy BMI or postpartum weight retention or measures of glucose homeostasis and insulin tolerance at postpartum was not significant (see the “Methods” section), it was not considered as a confounder in the further analysis. A flow chart of sample selection and analysis steps of this study is provided in Additional file 1: Fig. S1.

**Table 1:** Demographic, anthropometric and clinical characteristics of the study participants

	All (*N* = 976)		Trio subjects (*N* = 263)					
Variable	Preconception		Preconception		Pregnancy			Postpartum
	*n*	Mean (Std) or %	*n*	Mean (Std) or %	*n*	Mean (Std) or %	*n*	Mean (Std) or %
Ethnicity								
Chinese	705	72.23%	205	77.95%	205	77.95%	205	77.95%
Malay	147	15.06%	34	12.93%	34	12.93%	34	12.93%
Indian	88	9.02%	15	5.70%	15	5.70%	15	5.70%
Others	36	3.69%	9	3.42%	9	3.42%	9	3.42%
Education								
Below university	363	37.19%	74	28.14%	74	28.14%	74	28.14%
University & above	612	62.70%	189	71.86%	189	71.86%	189	71.86%
Missing	1	0.10%	**---**	**---**	**---**	**---**	**---**	**---**
Parity								
Nulliparous	630	64.55%	162	61.60%	162	61.60%	162	61.60%
Parous	344	35.25%	101	38.40%	101	38.40%	101	38.40%
Missing	2	0.20%	**---**	**---**	**---**	**---**	**---**	**---**
Age at recruitment (years)	976	30.78 (3.73)	263	30.06 (3.26)	263	30.06 (3.26)	263	30.06 (3.26)
Pre-pregnancy BMI (kg/m^2^)	970	23.78 (5.25)	262	23.03 (4.40)	262	23.03 (4.40)	262	23.03 (4.40)
Breastfeeding^a^								
Yes	**---**	**---**	**---**	**---**	**---**	**---**	229	87.07%
No	**---**	**---**	**---**	**---**	**---**	**---**	24	9.13%
Missing	**---**	**---**	**---**	**---**	**---**	**---**	10	3.80%
Weight (kg)	971	60.71 (13.71)	262	58.75 (11.4)	262	66.66 (11.62)	255	61.69 (11.85)
Gestational weight gain^b^ (kg)	**---**	**---**	---	---	261	7.77 (3.88)	---	---
Weight loss^c^ (kg)	**---**	**---**	---	---	---	---	253	4.84 (3.54)
Postpartum weight retention^d^ (kg)	**---**	**---**	---	---	---	---	253	2.95 (4.37)
Total cholesterol (mmol/L)	963	4.82 (0.83)	241	4.69 (0.78)	241	6.37 (0.96)	241	5.03 (0.86)
Triglyceride (mmol/L)	962	0.90 (0.50)	241	0.79 (0.35)	241	2.02 (0.63)	241	0.78 (0.36)
HDL cholesterol (mmol/L)	963	1.43 (0.30)	241	1.46 (0.28)	241	1.99 (0.35)	241	1.59 (0.32)
LDL cholesterol (mmol/L)	962	2.97 (0.74)	241	2.87 (0.7)	241	3.46 (0.81)	241	3.09 (0.78)
Fasting glucose (mmol/L)	973	4.81 (0.67)	249	4.73 (0.34)	249	4.27 (0.35)	249	4.56 (0.38)
2-h post-load glucose (mmol/L)	965	5.98 (1.95)	226	5.59 (1.17)	226	6.67 (1.29)	226	5.66 (1.05)
Fasting insulin (mU/mL)	974	7.12 (6.35)	243	5.7 (3.61)	243	7.07 (3.78)	243	4.80 (3.2)
HOMA-IR	971	1.57 (1.66)	240	1.19 (0.78)	240	1.35 (0.76)	240	1.00 (0.72)
HbA1c (%)	973	5.13 (0.42)	249	5.06 (0.27)	249	4.79 (0.29)	249	5.10 (0.28)

*BMI* body mass index, *HDL* high-density lipoprotein, *LDL* low-density lipoprotein, *HOMA-IR* homeostatic model assessment for insulin resistance, *HbA1c* glycated haemoglobin

^a^Exclusive breastfeeding at 3 months postpartum

^b^Gestational weight gain: the difference between preconception weight (reference) and the weight at pregnancy (26–28 weeks of gestation)

^c^Weight loss: the difference between the weight at pregnancy (26–28 weeks of gestation) and the weight at 3 months postpartum (reference)

^d^Postpartum weight retention: the difference between preconception weight (reference) and the weight at 3 months postpartum

### Adaptations in lipid metabolism to pregnancy

#### The lipidomic differences between pre-pregnant and non-pregnant women at preconception

Within the study subjects, only less than half of the women got pregnant within 12 months of recruitment. Characteristics of non-pregnant and pre-pregnant subjects (see the “Methods” section) at preconception are provided in Additional file 2: Table S1C. We observed that non-pregnant subjects were older (~1.4 years) and had lower educational attainment with higher cardiometabolic risk (i.e. higher BMI and concentrations of cholesterol, triglyceride and glycaemic traits) than pre-pregnant subjects. The lipidomic differences between non-pregnant and pre-pregnant women are shown in Additional file 2: Table S1D. We observed that 87 lipid species showed differential concentrations between two groups based on nominal *p*-value cut-off (*P* < 0.05; no lipid with *P*_adj_ < 0.05). Compared to non-pregnant subjects, pre-pregnant subjects have lower concentrations in the lipid species from triacylglycerols, phospholipids containing n-6 fatty acids (i.e. 18:2, 20:3, 20:4 and 22:4) and cholesteryl ester lipids. TG (54:1) showed the most significant difference between two groups (*P *= 3.41E−03 and 14.81% lower concentration in pre-pregnant compared to non-pregnant subjects).

#### Longitudinal analysis of plasma lipidome in response to the change in pregnancy status

To understand the modulation of plasma lipidomic profiles in a longitudinal manner from preconception to postpartum, three comparative analyses (pregnancy vs. preconception, pregnancy vs. postpartum and postpartum vs. preconception) were performed by paired *t*-test using trio subjects (Additional file 2: Table S1E and Fig. 1C, D). We observed that 66.4% of lipid species (FC > 1.5: 36.9% and 0 < FC ≤ 1.5: 29.5%) increased and 25.1% of lipid species (FC < −1.5: 10.4% and −1.5 ≤ FC < 0: 14.7%) decreased in concentration in pregnancy as compared to preconception (Fig. 1C, D). The most significant increase was observed in phosphatidylethanolamine and phosphatidylinositol lipid species followed by triacylglycerols, diacylglycerols and alkyl-diacylglycerols. The majority of the cholesteryl esters as well as free cholesterol increased in pregnancy with selective decrease in a few cholesteryl ester species (i.e. CE(20:4) and CE(20:5)). At the same time, most lysophospholipids and acylcarnitines decreased in pregnancy. However, a few saturated lysophospholipids including LPE(16:0) showed increase in concentration in pregnancy. Most sphingolipids showed significant differences but with small fold changes (|FC| ≤ 1.5) except that of deoxy-ceramide, ceramide-1-phosphate and several lipids from sphingomyelin, dehydroceramide and dihexosylceramide classes. A comparison between pregnancy and postpartum showed similar trends to those between pregnancy and preconception (*R*^2 ^= 0.97 in effect size). However, we observed a difference in the percentage of significant lipids between these two comparison results. Around 56.7% of lipid species (FC > 1.5: 32.5% and 0 < FC ≤ 1.5: 24.2%) increased and 33.5% of lipid species (FC < −1.5: 12.2% and −1.5 ≤ FC < 0: 21.3%) decreased in pregnancy compared to postpartum. Although there was a significant increase in 59.5% of lipid species (FC > 1.5: 0.4% and 0 < FC ≤ 1.5: 59.1%) and a decrease in 9.1% of lipid species (FC < −1.5: 0.0% and −1.5 ≤ FC < 0: 9.1%) at postpartum compared to preconception, most of these lipid species had small fold changes (|FC| ≤ 1.5) except that of three dehydrocholesterol lipid species DE(18:2) (FC = 1.53), DE(20:4) (FC = 1.54) and DE(20:5) (FC = 1.69).

#### Patterns of lipid changes across preconception, pregnancy and postpartum

**Fig. 2:**
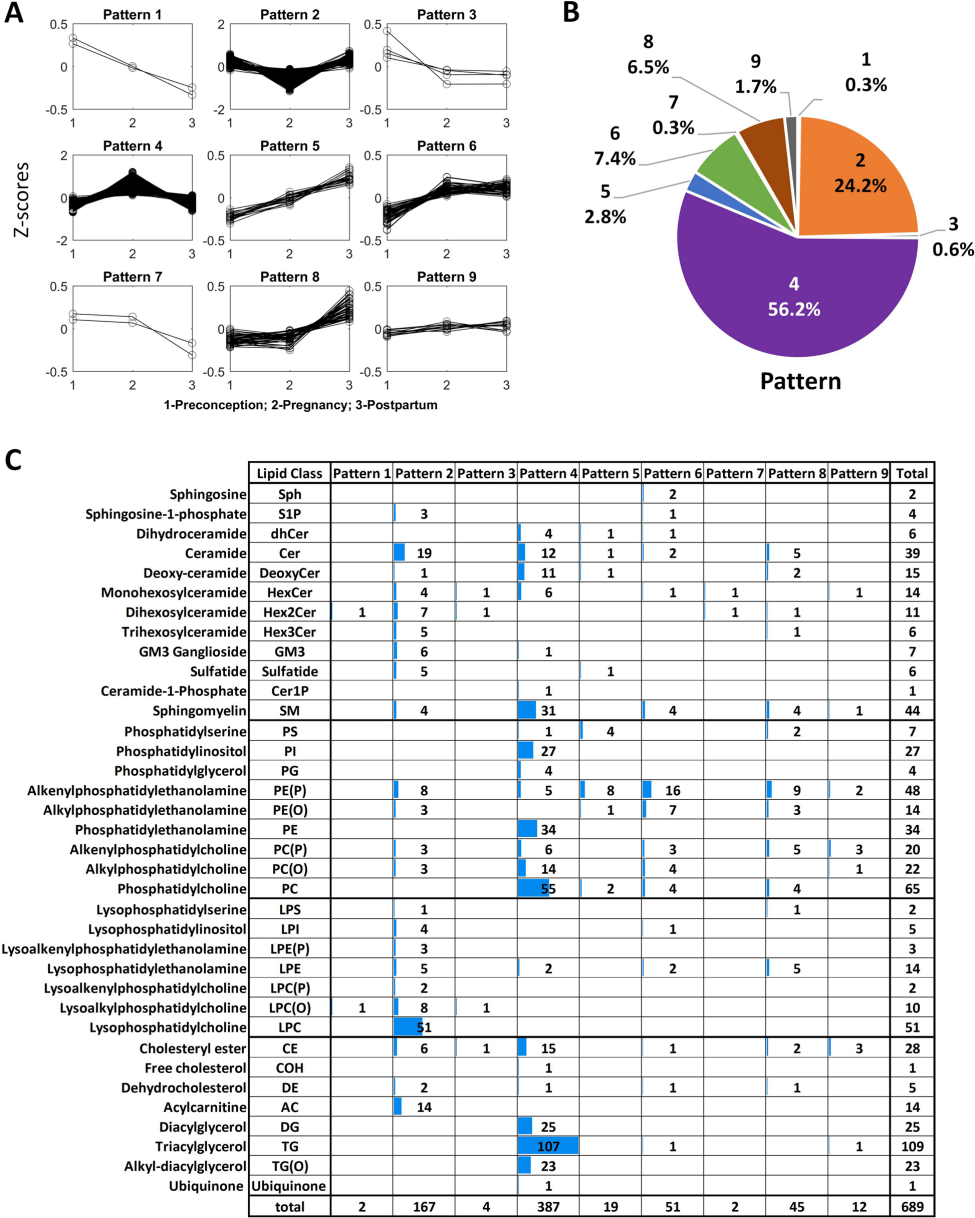
Patterns of longitudinal changes in plasma lipidomic profiles from preconception to postpartum using trio subjects (*n *= 263). **A** Nine patterns of lipid change profiles (*z*-scores of log-transformed lipidomic data). **B** Pie chart of nine patterns. **C** Distribution of nine patterns in lipid classes

All measured lipid species could be classified into nine main patterns (see the “Methods” section and Fig. 2) based on the results of two comparative lipidomic studies (pregnancy vs. preconception and pregnancy vs. postpartum) as the lipidomic profiles at postpartum and preconception were similar (Fig. 1B, C). The majority of the lipid species belong to pattern 4 (56.2%, increased in pregnancy and returned to the preconception concentrations at postpartum) and pattern 2 (24.2%, decreased in pregnancy and returned to the preconception concentrations at postpartum). In pattern 4, phospholipids species from phosphatidylethanolamine and phosphatidylinositol classes showed the most significant increase in pregnancy followed by an overall increase in glycerolipids lipids including triacylglycerols, alkyl-diacylglycerols and diacylglycerols. The lipid species in pattern 2 showed a decrease in pregnancy, represented by lysophospholipids, acylcarnitine, very long-chain ceramides and complex sphingolipids. Interestingly, the elevated lipid species (66.4%) from preconception to pregnancy (Fig. 1D) can be classified into patterns 4, 5 and 6 (Fig. 2A) based on their concentrations at postpartum. Amongst these, 56.2% of lipid species (pattern 4) decreased in concentration at postpartum, 2.8% (pattern 5) were still present at higher concentration at postpartum and 7.4% (pattern 6) were at similar concentrations as that of pregnancy at postpartum. Pattern 5 consisted of several lipids from alkenylphosphatidylethanolamine (PE-plasmalogens) and phosphatidylserine. Pattern 6 was mainly represented by a few lipid species from ether-linked phospholipid classes. Comparison of lipid profile between the three time points is shown in Additional file 2: Table S1B. The results showed that the concentrations of total cholesterol, high-density lipoprotein-cholesterol (HDL-C), low-density lipoprotein-cholesterol (LDL-C) and triglyceride (TG) were elevated during pregnancy and decreased at postpartum. Compared to the concentrations at preconception, the concentrations of total cholesterol, HDL-C and LDL-C at postpartum were still slightly higher, but the concentration of TG at postpartum had returned to the preconception concentration. The changes in lipid profile from preconception to postpartum followed the trend of pattern 4.

### Lipid signatures linked with cardiometabolic risk traits

#### Associations of body weight changes with the changes in lipidomic profiles

**Fig. 3:**
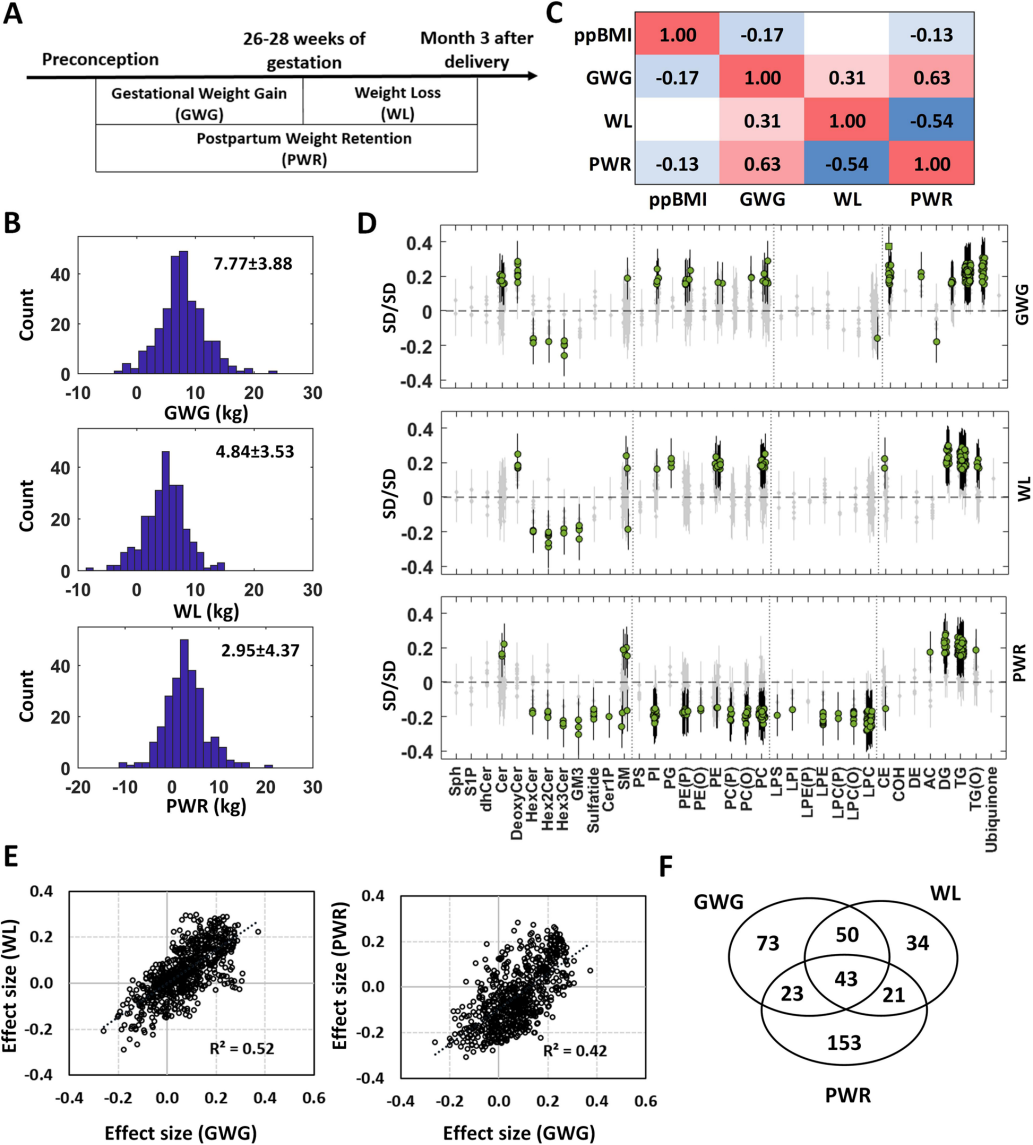
Associations of body weight changes with the changes in lipidomic profiles. **A** Gestational weight gain (GWG), weight loss (WL) and postpartum weight retention (PWR). **B** Histograms of GWG (*n* = 261), WL (*n* = 253) and PWR (*n* = 253). **C** Pairwise Pearson correlation (R) heat map between pre-pregnancy BMI (ppBMI) and body weight changes. **D** Forest plots (error bar: 95% CI) of three association results. Diamond—*P*_adj_ ≥ 0.05 (grey), circle—*P*_adj_ < 0.05 and square—*P*_adj_ < 1.00E−5. **E** Scatter plots of effect sizes (SD/SD). Effect size is SD change in log_2_FC for one SD increase in body weight change. **F** Venn diagram of significant lipid species in three analyses

As both lipidomic profiles and body weights undergo significant changes between pregnant and non-pregnant states, the association analyses between the changes in lipidomic profiles (log_2_FC in Fig. 1C) and body weight changes (Fig. 3A) were studied using trio subjects. Gestational weight gain (GWG: 7.77 ± 3.88 kg), weight loss (WL: 4.84 ± 3.53 kg) and postpartum weight retention (PWR: 2.95 ± 4.37 kg) were normally distributed (Fig. 3B). Pairwise Pearson correlation coefficients (R) of body weight changes and pre-pregnancy BMI (ppBMI) are provided in Fig. 3C. GWG was positively associated with PWR (*R *= 0.63) and WL (*R *= 0.31). PWR was negatively associated with WL (*R *= −0.54). The results of lipidomic analysis on body weight changes are presented in Fig. 3D and Additional file 3: Table S2A. Higher GWG resulted in higher concentrations in lipid species from ceramide, deoxy-ceramide, most of phospholipid and neutral lipid classes, but lower concentrations in glycosphingolipids including monohexosylceramide, dihexosylceramide and trihexosylceramide species (189 of 689, 27.4%). There were 148 significant lipid species (21.8%) associated with WL. Amongst these, 93 lipids (62.8%) overlapped with the results in the GWG analysis. We observed similarities in neutral lipids (73 glycerolipids and 2 cholesteryl esters: CE(16:1) and CE(20:3)), phospholipids (7 lipids), deoxy-ceramide (6 lipids), glycosphingolipids (4 lipids) and 1 sphingomyelin lipid (SM(d18:2/14:0)), that led to moderate correlation (Fig. 3E, *R*^2 ^= 0.52) between the effect sizes of the GWG and WL analyses. Ceramide, dehydrocholesterol and ether-link phospholipid lipids showed significant association with GWG, but no association with WL. As compared to the GWG analysis, a higher number of lipid species (240 of 689, 34.8%) were associated with PWR. Similar trends were found for neutral lipid classes (triacylglycerol and diacylglycerol), ceramides and glycosphingolipids; hence, the effect sizes of the GWG and PWR analyses still showed positive association (Fig. 3E, *R*^2 ^= 0.42). Sphingomyelins showed divergent trends of associations with PWR, depending on the composition of individual species. Phospholipids and lysophospholipids showed unique features in the PWR analysis as the change in lipid concentration decreased with increase in PWR. Forty-three lipid species showed consistent trends in these three body weight change association analyses (Fig. 3F and Additional file 3: Table S2A). These included the lipid species from sphingomyelin (SM(d18:2/14:0)), dihexosylceramide (Hex2Cer(d18:1/24:1)), trihexosylceramide (Hex3Cer(d18:1/24:0) and Hex3Cer(d18:1/24:1)), diacylglycerol (DG(16:0_16:1), DG(16:0_22:6) and DG(18:1_20:4)) and triacylglycerol (36 lipids) primarily those containing unsaturated fatty acids. The associations between lipid profile and body weight changes are provided in Additional file 3: Table S2B. GWG was not associated with changes in lipid profile whereas WL was only associated with change in TG (*β *= 0.22, *P *= 7.38E−04). PWR was significantly associated with changes in LDL-C (*β *= 0.18, *P *= 7.48E−03), HDL-C (*β *= −0.37, *P *= 1.06E−08) and TG (*β *= 0.21, *P *= 1.94E−03). In addition, it was noted that ppBMI was significantly negatively associated with changes in total cholesterol, LDL-C and TG concentrations, but positively associated with change in HDL-C concentration from pregnant to non-pregnant states (Additional file 3: Table S2B). No significant association was observed with the changes in lipid profile between preconception and postpartum for ppBMI. Similar results were also found in the lipidomic analysis for ppBMI in the body weight change studies (Additional file 3: Table S2A). A number of lipid species showed a significant association of ppBMI with changes in lipidomic profiles between pregnancy and preconception (136 lipids) and between pregnancy and postpartum (176 lipids), respectively. More than 94% of significant lipids showed a negative association in these two analyses. No lipids showed a significant association of ppBMI with changes in lipidomic profiles between preconception and postpartum.

#### Lipid signatures of pre-pregnancy BMI at preconception, pregnancy and postpartum

**Fig. 4:**
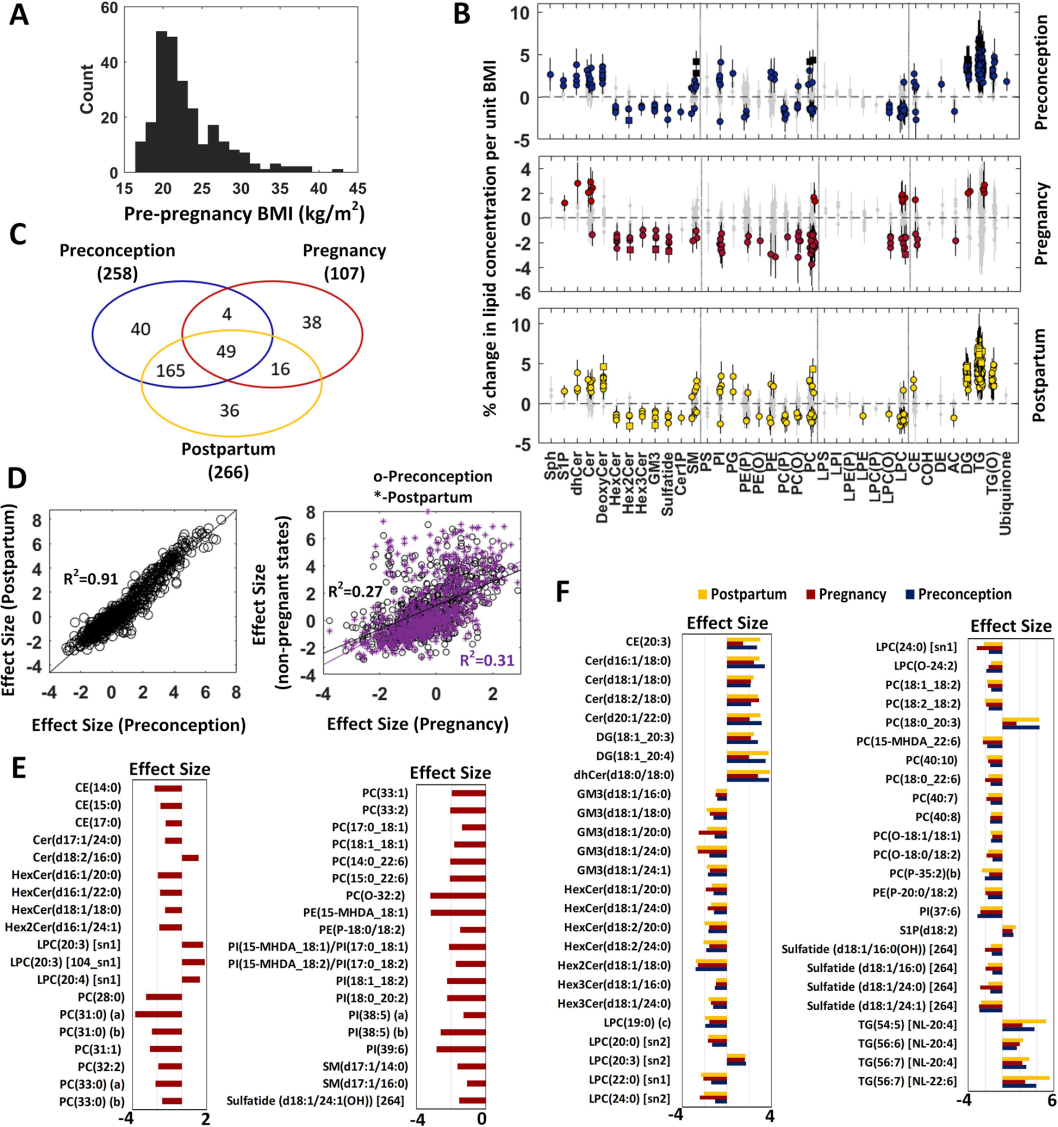
Association of pre-pregnancy BMI (ppBMI) with plasma lipidomic profiles at preconception, pregnancy and postpartum using trio subjects (*n *= 252). **A** Histogram of ppBMI. **B** Forest plots (error bar: 95% CI) of the ppBMI association studies at three time points. Diamond—*P*_adj_ ≥ 0.05 (grey), circle—*P*_adj_ < 0.05 and square—*P*_adj_ < 1.00E−5. **C** Venn diagram of significant lipid species in three studies. **D** Scatter plots of effect sizes (% change in lipid concentration per unit BMI). **E** Thirty-eight unique pregnancy-related lipid signatures. **F** Forty-nine common lipid signatures in three studies

The distribution of ppBMI (mean = 23.03 kg) of the study subjects is represented in Fig. 4A. The association analyses of ppBMI with lipidomic profiles from preconception to postpartum were studied using trio subjects (Additional file 4: Table S3 and Fig. 4B, C). We observed similarities in association of lipidomic profiles at two non-pregnant states (preconception and postpartum). Strong correlation (*R*^2 ^= 0.91) of their effect sizes was observed (Fig. 4D). Glycerolipids (triacylglycerols, alkyl-diacylglycerols, diacylglycerols) and sphingolipids (sphingosine-1-phosphate, dihydroceramide, ceramide and deoxy-ceramide) showed positive associations whereas glycosphingolipids, sulfatides, ceramide-1-posphate and lysophospholipids (excluding LPC(20:3)) showed negative associations with ppBMI. Phospholipids and sphingomyelin classes showed a divergent trend in their associations with ppBMI. Compared to non-pregnant states, the lipid signatures of ppBMI at pregnancy presented some distinct features. Sphingomyelin and phospholipids (excluding PC(18:0_20:3) and PC(18:0_20:4)) classes showed negative association with ppBMI. Smaller number of neutral lipids (triacylglycerols and diacylglycerols) was found to be associated with ppBMI. Weak correlation (*R*^2^ = ~0.3) of their effect sizes between pregnant and non-pregnant states was observed (Fig. 4D). Apart from a noticeable overlap (>80%) in the associations of lipids species at preconception and postpartum with ppBMI, 38 lipids (Fig. 4E) showed unique pregnancy-related association with ppBMI and 49 lipids species (Fig. 4F) were common in three analyses (Fig. 4C). The 38 unique pregnancy-related lipids included phospholipids with odd chain or branched chain containing fatty acids, and lower carbon number and low degree of unsaturation containing fatty acid species (Fig. 4E). In the 16 overlapping lipids between pregnancy and postpartum (Fig. 4C), we also found that five phospholipids with odd chain (PC(17:0_18:2) and PC(17:1_18:2)) and branched chain (PC(15-MHDA_18:1), PC(15- MHDA_18:2) and PE(15-MHDA_18:2)) fatty acid displayed significant association. From the 49 common lipid species in three analyses, ceramide, diacylglycerol and triacylglycerol species showed a positive association whereas glycosphingolipid, phospholipid and lysophospholipid species showed a negative association. Interestingly, the lipid species containing dihomo-γ-linolenic acid (DGLA) and arachidonic acid (ARA) including CE 20:3, DG(18:1_20:3), DG(18:1_20:4), LPC(20:3), PC(18:0_20:3), and TG(56:6) [NL-20:4] displayed consistent positive association with ppBMI (Fig. 4F).

#### Relationship of lipidomic profiles with measures of glucose homeostasis and insulin resistance

**Fig. 5:**
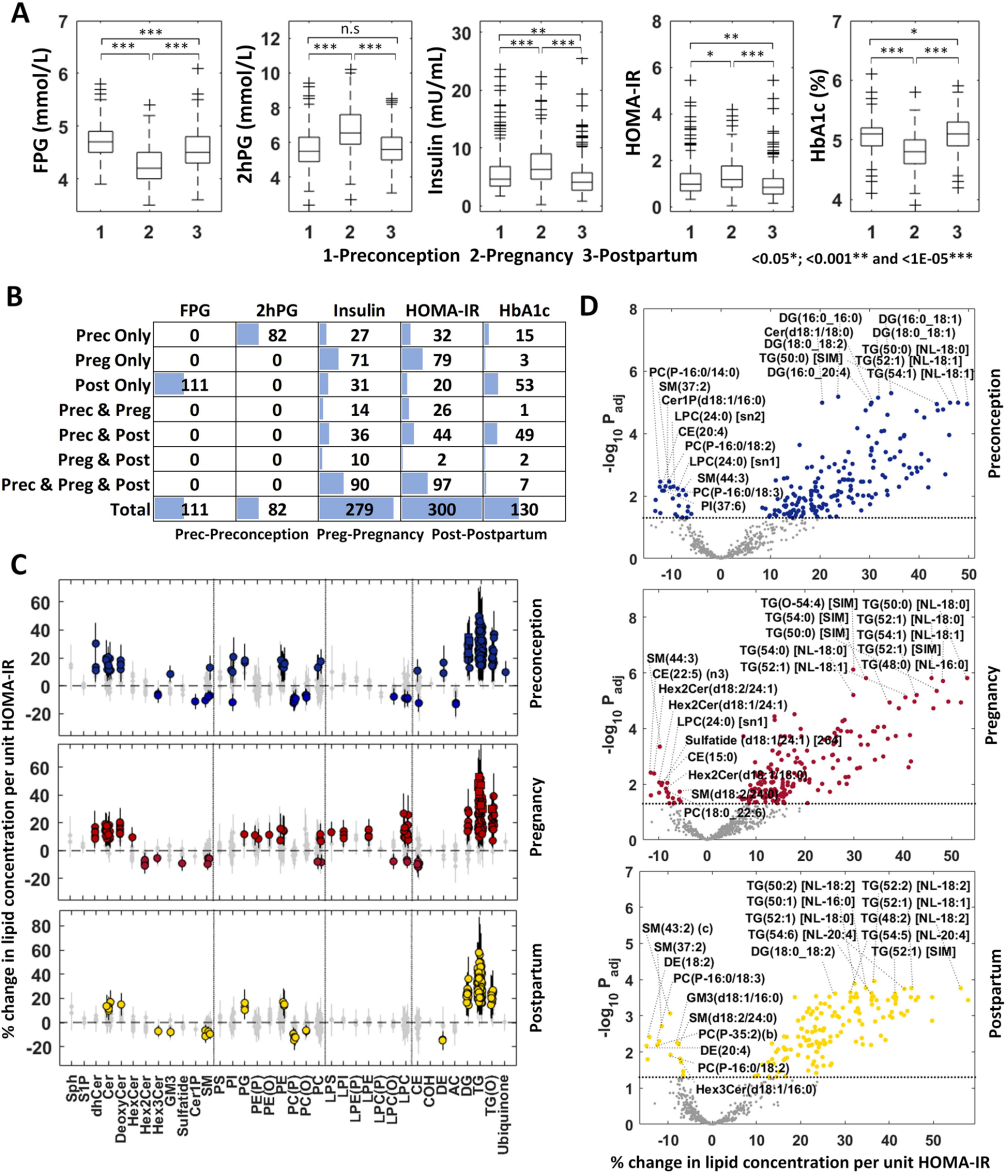
Association of measures of glucose homeostasis and insulin resistance with plasma lipidomic profiles at preconception, pregnancy and postpartum using trio subjects. **A** Box plots of fasting glucose (*n* = 249), 2-h post-load glucose (*n* = 226), fasting insulin (*n* = 243), HOMA-IR (*n* = 240) and HbA1c (*n* = 249) at three time points. The *p*-values are calculated by paired t-test. **B** Number of significant lipid species at three time points (*P*_adj_ < 0.05). **C**, **D** Forest plots (error bar: 95% CI) and volcano plots of the HOMA-IR studies at three time points. Diamond—*P*_adj_ ≥ 0.05 (grey), circle—*P*_adj_ < 0.05 and square—*P*_adj_ < 1.00E−5 in forest plots. Top 10 lipid species with positive and negative associations are labelled in (**D**). The horizontal dotted line in **D** indicates *P*_adj_ = 0.05

Fasting plasma glucose (FPG), 2-h post-load glucose (2hPG), fasting insulin concentrations, HOMA-IR and glycated haemoglobin (HbA1c, %) at preconception, pregnancy and postpartum were investigated using trio subjects to understand the longitudinal associations with the plasma lipidomic profiles. The pairwise Pearson correlation heat map, data distributions and pairwise scatter plots are illustrated in Additional file 1: Fig. S2-5. The boxplots at three time points are provided in Fig. 5A. We observed that FPG concentration and HbA1c level decreased in pregnancy and then increased at postpartum. The FPG concentration at postpartum was lower than that at preconception while HbA1c level at postpartum was slightly higher than the preconception concentration (Additional file 2: Table S1B). The 2hPG, insulin and HOMA-IR concentrations increased during pregnancy and decreased at postpartum. 2hPG concentration at postpartum was near to that at preconception while insulin and HOMA-IR concentrations at postpartum were significantly lower than those at preconception (Additional file 2: Table S1B). Their association results with plasma lipidomic profiles at three time points are provided in Additional file 5: Table S4A-G.

For FPG, no significant associations with lipid species (*P*_adj_ < 0.05) were found at preconception and pregnancy, but 111 lipid species showed significant associations at postpartum (Additional file 5: Table S4A). Glycerolipids (triacylglycerols, alkyl-diacylglycerols and diacylglycerols), ceramide and several lipids from deoxy-ceramide (Cer(m17:0/22:0)), sphingosine (Sph(d18:2)) and phospholipids (PE(18:0_18:1) and PG(36:1)) were positively associated with FPG whereas only two lipids (DE(20:4) and PC(P-16:0/18:3)) showed negative association with FPG at postpartum (Fig. 5B and Additional file 1: Fig. S6A). Amongst these, triacylglycerols and diacylglycerols containing linoleic acid (i.e. TG(52:2) [NL-18:2] and DG(18:0_18:2)) showed the strongest association. Moderate correlation (*R*^2 ^= 0.65) of the effect sizes at two non-pregnant states (preconception and postpartum) was observed while weak correlation (*R*^2 ^= ~0.30) of the effect sizes was observed between pregnant and non-pregnant states (Additional file 1: Fig. S7). As no significant associations were found at preconception and pregnancy, we also investigated the overlapping lipid species based on nominal *p*-value cut-off (<0.05) at three time points (Additional file 1: Fig. S8A). The number of significant lipids at pregnancy (55 lipids) was much less than that at preconception (196 lipids) and postpartum (175 lipids). Twenty-four common lipids, which consisted of triacylglycerols with lower carbon number and low degree of unsaturation (containing 16:0, 18:0 and 18:1 fatty acids), very long chain ceramides (i.e. Cer(d16:1/22:0)) and deoxy-ceramides (Cer(m17:0/22:0) and Cer(m17:0/23:0)), showed consistent positive association with FPG at three time points (Additional file 1: Fig. S8A).

In the 2hPG study, we found 82 lipids with significant association at preconception, but no significant associations were found at pregnancy and postpartum (Fig. 5B and Additional file 5: Table S4B). Moderate correlation (*R*^2 ^= 0.47) of effect sizes was observed between preconception and pregnancy whereas very weak correlation (*R*^2 ^= 0.18) of effect sizes were observed between postpartum with the other two time points (Additional file 1: Fig. S7). Neutral lipids (triacylglycerols, alkyl-diacylglycerols, diacylglycerols, CE(20:3) and DE(18:1)) and four lipids from phospholipids (PC(18:0_20:3), PE(18:0_22:6), PI(18:0_20:3) and PG(36:1)) were positively associated with 2hPG while only one lipid (SM(37:2)) showed negative association with 2hPG at preconception (Additional file 5: Table S4B and Additional file 1: Fig. S6B). Triacylglycerols and diacylglycerols containing palmitic acid (16:0) showed the strongest association. As no significant associations were found at pregnancy and postpartum, we also studied the overlapping lipid species based on nominal *p*-value cut-off (<0.05) at the three time points (Additional file 1: Fig. S8B). The numbers of significant lipids at pregnancy (50 lipids) and postpartum (15 lipids) were much lower than that at preconception (189 lipids). No overlapping lipids were found at three time points.

In this study, 57 mothers were diagnosed with gestational diabetes mellitus (GDM) during pregnancy. To assess lipid signatures of GDM, associations of GDM status (GDM vs. Normal) with plasma lipidomic profiles at three time points were investigated (Additional file 5: Table S4C). We found that 37 lipid species at preconception were associated with GDM status based on nominal *p*-value cut-off (<0.05) (Additional file 5: Table S4D and Additional file 1: Fig. S8C). Seven lipids comprising lysophosphatidylcholine (LPC(20:1), lysoalkylphosphatidylcholine (LPC(O-24:2)), sphingomyelin (SM(40:3) and SM(44:3)) and phosphatidylinositol (PI(18:1_18:2), PI(18:0_20:2) and PI(18:0_22:5) (n6)) showed negative associations. Thirty lipids from triacylglycerol and alkyl-diacylglycerol classes with low degree of fatty acid unsaturation (19 TG and 6 TG(O) lipids), diacyglycerol (DG(16:0_16:0)), ceramide (Cer(d18:1/23:0) and Cer(d19:1/23:0)) and deoxy-ceramide (Cer(m18:0/20:0) and Cer(m18:1/18:0)) classes showed a positive association. Amongst these, Cer(m18:0/20:0) showed the strongest positive association whereas SM(44:3) showed the strongest negative association. A total of 72 lipid species in pregnancy were associated with GDM status (Additional file 1: Fig. S8C). Except for two cholesterol ester species (CE(15:0) and CE(20:4)), all the other lipid species from triacylglycerol and alkyl-diacylglycerol with low degree of fatty acid unsaturation (45 TG and 12 TG(O)), diacylglycerol (8 species containing FA18:1, FA18:2 and FA18:3), ceramide (4 Cer) and deoxy-ceramide (Cer(m18:1/18:0)) classes showed higher concentrations in GDM subjects. Amongst these, 45 lipid species (28 TG, 4 TG(O), 8 DG, 4 Cer and CE(15:0)) were the unique pregnancy-related signatures. We also observed that 83 lipid species at postpartum were still associated with GDM status (Additional file 1: Fig. S8C). Positive associations were observed in triacylglycerol and alkyl-diacylglycerol species, and negative associations were found in multiple lipid classes including acylcarnitine, cholesterol ester, sphingolipids and lysophosphatidylcholine. Amongst these, TG(58:8) showed the strongest positive association whereas AC(14:1) showed the strongest negative association. Ten common lipids including triacylglycerols (containing FA18:0, FA18:1 and FA17:1) and alkyl-diacylglycerols (containing FA17:1) showed consistent positive trends at the three time points (Additional file 1: Fig. S8C). The profiles of six selected species between GDM and non-GDM groups were shown in Additional file 1: Fig. S8D.

Fasting plasma insulin was highly correlated with HOMA-IR (*R *= 0.99, Additional file 1: Fig. S2). Their association results with lipidomic profiles were also very similar (Additional file 5: Table S4E-F, Fig. 5C and Additional file 1: Fig. S9A). As their effect size plots showed very high correlation (*R*^2 ^≥ 0.97, Additional file 1: Fig. S9B), only the details of the HOMA-IR results are discussed in this section. Amongst the significant HOMA-IR-associated lipid species (*P*_adj_< 0.05) at preconception (199 lipids), pregnancy (204 lipids) and postpartum (163 lipids), 97 common lipids were found in three analyses (Fig. 5B). Ninety-five lipids including triacylglycerol, alkyl-diacylglycerol, diacylglycerol, ceramide classes and several lipid species (Cer(m17:0/22:0), PE(18:0_18:1) and PG(36:1)) showed consistent positive association whereas only two lipids (SM(43:2) and Hex3Cer(d18:1/16:0)) showed negative association with HOMA-IR. Triacylglycerol and diacylglycerol containing 16:0, 18:0, 18:1 and 18:2 fatty acids showed the strongest positive association with HOMA-IR (Fig. 5D). From the 79 unique pregnancy-related lipids (Fig. 5B), lysophospholipids (30 lipids excluding LPC(O-24:1)) showed a positive association with HOMA-IR in pregnancy. We observed similarities in the association of lipidomic profiles at two non-pregnant states (preconception and postpartum) with strong correlation (*R*^2 ^= 0.85) of their effect sizes (Additional file 1: Fig. S7A). A moderate correlation of effect sizes (*R*^2^ = ~0.60) was observed between pregnant and non-pregnant states (Additional file 1: Fig. S7B).

In the HbA1c study, we observed more significant lipids at preconception (72 lipids) and postpartum (111 lipids) than those in pregnancy (13 lipids) as shown in Additional file 5: Table S4G. A large number of glycerolipids (triacylglycerol, alkyl-diacylglycerol, diacylglycerol) and ceramide lipids were associated with lipidomic profiles in non-pregnant states (Additional file 1: Fig. S6C). Compared to the results at preconception, more phospholipids (phosphatidylethanolamine and phosphatidylglycerol) showed significant association at postpartum. Hex2Cer(d18:2/16:0), LPC(O-24:1) and LPC(O-24:2) showed association with HbA1c only in pregnancy. Seven common lipids including six triacylglycerol lipids containing 18:0 and 18:1 fatty acids and one deoxy-ceramide lipid (Cer(m17:0/24:0)) were found in three analyses (Fig. 5B). A moderate correlation of effect sizes (*R*^2 ^= 0.73) was observed between two non-pregnant states (Additional file 1: Fig. S7A), and a slightly low correlation of effect sizes (*R*^2 ^= ~0.65) was observed between pregnant and non-pregnant states (Additional file 1: Fig. S7B).

#### Comparative assessment of lipid signatures of measures of glucose homeostasis and insulin resistance at preconception

**Fig. 6:**
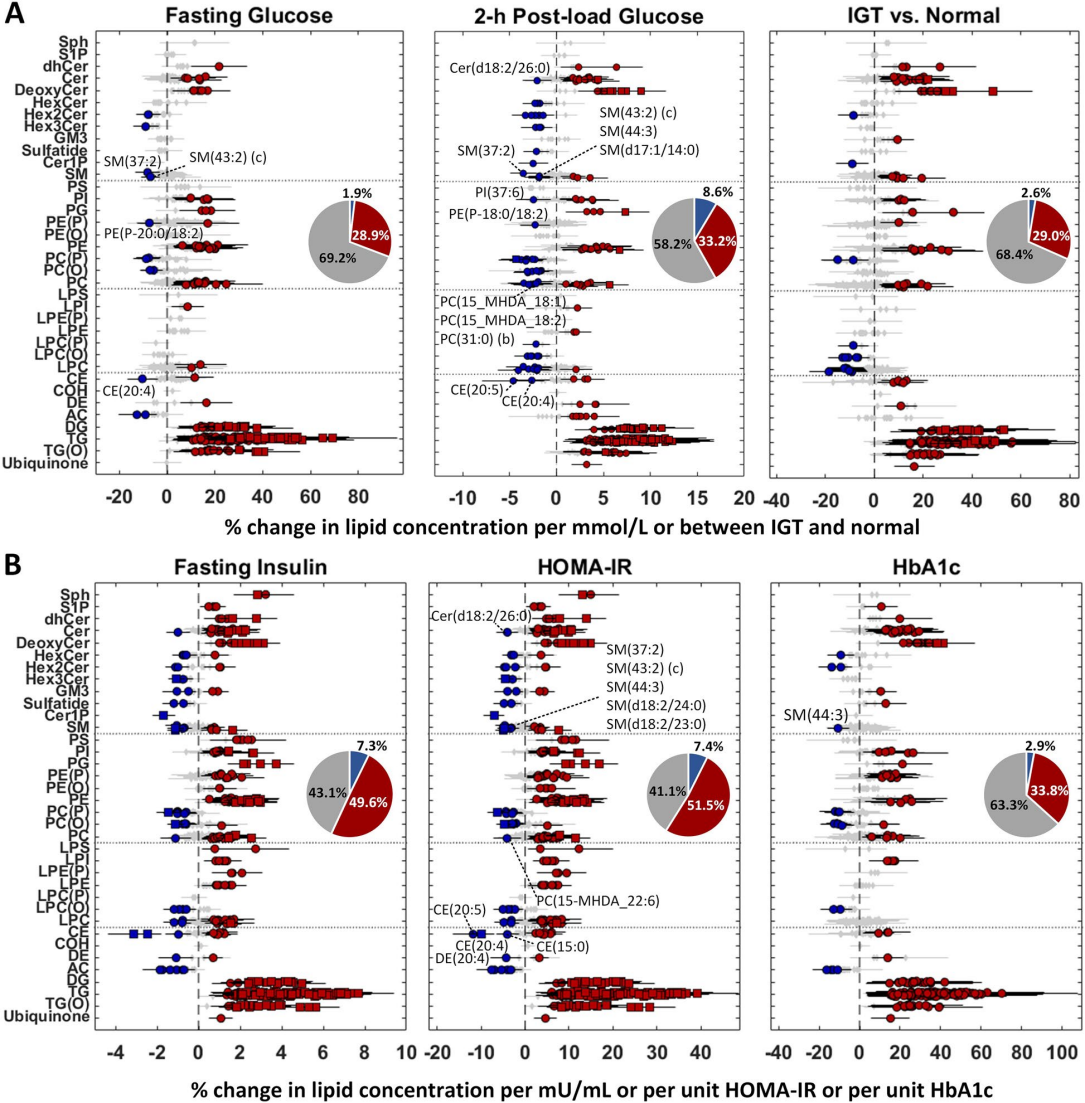
Association of measures of glucose homeostasis and insulin resistance with plasma lipidomic profiles at preconception (*n* = 936). **A** Forest plots (error bar: 95% CI) of fasting glucose concentration (mmol/L), 2-h post-load glucose concentrations (mmol/L) and impaired glucose tolerance status (86 IGT vs. 850 Normal). **B** Forest plots (error bar: 95% CI) of fasting insulin concentration (mU/mL), HOMA-IR and HbA1c (%). Diamond—*P*_adj_ ≥ 0.05 (grey), circle—*P*_adj_ <0.05 and square—*P*_adj_ <1.00E−5. Pie chart—percentage of lipids with significant (positive: red and negative: blue) or insignificant (grey) associations

As the preconception period offers an opportunity for the assessment of metabolic health before pregnancy, we carried out detailed characterization of the lipid signatures of FPG, 2hPG, impaired glucose tolerance (IGT) status, fasting insulin concentrations, HOMA-IR and HbA1c (*n *= 936). The data distributions and pairwise correlations are shown in Additional file 1: Fig. S10A-B. Forest plots of these six studies are illustrated in Fig. 6. HOMA-IR exhibited the highest percentage of significant lipids (58.9%), followed by insulin (56.9%), 2hPG (41.8%), HbA1c (36.7%), IGT status (31.6%) and FPG (30.8%). We observed very high similarity of association results between insulin and HOMA-IR (*R*^2 ^= 1.0 in effect size, Additional file 1: Fig. S10C). The results of lipids associated with IGT status (defined as an elevated 2hPG with normal FPG level) were very similar (*R*^2 ^= 0.94 in effect size, Additional file 1: Fig. S10C) to those of 2hPG but with a smaller number of significant lipids. Therefore, only the four studies (FPG, 2hPG, HOMA-IR and HbA1c) were used for comparative assessment (Additional file 6: Table S5 and Additional file 1: Fig. S10D). Interestingly, 157 common lipids with consistent trends were found in four studies. Apart from the three lipids (PC(O-34:1), PC(O-18:1/18:1) and Hex2Cer(d18:2/24:1)), 154 lipids from triacylglycerol, alkyl-diacylglycerol, diacylglycerol, ceramide, deoxy-ceramide classes and nine lipids (CE(16:1), dhCer(18:0/18:0), PE(16:0_16:1), PE(18:0_18:2), PE(18:0_20:3), PE(18:0_22:6), PG(36:1), PI(16:0_16:1) and PI(16:0_20:4)) showed a positive association in the four studies. Amongst these, DG(16:0_16:0), TG(50:0)[NL-18:0], Cer(d18:1/18:0) and Cer(m18:0/20:0) showed the strongest association in their classes. Only one ceramide species (Cer(d18:2/26:0)) showed a negative association with 2hPG and HOMA-IR. Sphingomyelin lipids with either a d17:1 or d18:2 long chain base were negatively associated with four measures. SM(44:3) showed the strongest negative association with four measures. For phospholipids, most of lipids showed strong positive association except alkylphosphatidylcholine, alkenylphosphatidycholine, phosphatidylcholine containing odd- or branched-chain fatty acids and a few other lipids including (PE(P-18:0_18:2) and PI(37:6)) (Fig. 6). PG(36:1) showed the strongest positive association within the phospholipids. A higher number of lysophospholipids were associated with 2hPG and HOMA-IR than FPG and HbA1c. LPC(O-24:2) showed the strongest association with four measures in lysophospholipids. In cholesteryl ester, only CE(15:0), CE(20:4) and CE(20:5) showed a negative association while others showed a positive association with four measures. Acylcarnitines were negatively associated with FPG, HOMA-IR and HbA1c, but positively associated with 2hPG (Fig. 6). Last, the above results were compared to the association results at preconception using trio subjects as described in the previous section. We observed their effect sizes were highly correlated (FPG: *R*^2 ^= 0.82, 2hPG: *R*^2 ^= 0.87, insulin: *R*^2 ^= 0.89, HOMA-IR: *R*^2 ^= 0.92 and HbA1c: *R*^2 ^= 0.82) as illustrated in Additional file 1: Fig. S11.

## Discussion

This study is the first of its kind, to the best of our knowledge, to examine the changes in plasma lipidome from preconception through pregnancy to postpartum in childbearing women. We carried out detailed mapping of the changes in concentrations of diverse lipid species in response to pregnancy. Pregnancy represents both physiological and physical challenges that could provide a window into future maternal health outcomes. We identified plasma lipid species that are associated with the reversion and retention of the pregnancy-induced metabolic phenotypes with implications in postpartum health as well as an opportunity to intervene at the preconception stage. We further demonstrate the complex associations of maternal plasma lipidome with BMI, body weight changes and glycaemic traits in a longitudinal manner.

We observed that non-pregnant subjects (women who did not conceive) at preconception were at higher cardiometabolic risk (i.e. higher BMI and the concentrations of cholesterol, triglyceride and glycaemic traits) than pre-pregnant subjects. We identified differential concentrations of 87 lipid species in pre-pregnant compared to non-pregnant women. These included lipid species (i.e. triacylglycerols, phospholipids and cholesteryl ester) with higher concentrations in non-pregnant subjects that were also positively associated with BMI and glycaemic traits (Additional file 7: Table S6). These findings are consistent with the previous report on reduced fecundability of women with unhealthy metabolic health status in the same cohort [35].

Longitudinal lipidomic analyses illustrated the modulation of plasma lipidomic profiles from preconception to postpartum. Around 56% of lipid species increased in concentration in pregnancy as compared to both preconception and postpartum. These plasma lipidomic data were supplemented by the observation of an increase in total triglyceride and cholesterol concentrations as well. The most significant increase was observed in phospholipid classes PE and PI followed by an overall increase in TG concentrations. Although there was an increase in the concentrations of all the lipid species from PI and PE classes in pregnancy, upon consideration of the distribution of lipid species within the class, ARA containing species PI(16:0_20:4) and PI(18:0_20:4) as well as PC(18:0_20:4) and PE(18:0_20:4) were present at lower percentage of their respective total class concentrations as compared to both preconception and postpartum (Additional file 1: Fig. S12). At the same time, saturated, monounsaturated and DHA-containing phospholipid species were present at a higher percentage in pregnancy. The PUFA-PC are preferentially synthesized *via* three successive methylation steps by phosphatidylethanolamine *N*-methyltransferase (PEMT) in the liver [36]. We used the ratios of plasma concentration of 16:0-DHA PC to total PC as well as 18:0-DHA PC to total PC as surrogate markers for PEMT activity [37]. Both of these ratios were significantly increased in pregnancy, indicating an increased activity of PEMT (Additional file 1: Fig. S13A). Further, the decreased concentration of lysophospholipids as well as the total cholesteryl ester concentration suggests a decrease in the activity of phospholipases including lipoprotein-associated phospholipase A_2_ (Lp-PLA2) and lecithin-cholesterol acyltransferase (LCAT) in mid-late pregnancy. These observations were also supported by the decrease in LCAT and Lp-PLA2 enzyme indices derived from the lipidomics data. For these indices, CE/FC ratio was considered as proxy for LCAT activity (Additional file 1: Fig. S13B) whereas total LPC to PC and total LPE to PE ratios were used for PLA2 activity (Additional file 1: Fig. S13C). Together, these data provide insights into the modulation of phospholipid synthesis and metabolism leading to increased concentration of phospholipids in circulation for maternal-foetal transport. These phospholipids are hydrolysed by endothelial lipase (EL) of the placenta and support the transport of PUFA-containing lyso-PL *via* lysolipids transporters such as MFSD2a [38]. EL is primarily a phospholipase with high levels of phospholipase A1 activity [39] and releases lysophospholipids with *sn2* fatty acid chain intact that is often occupied by PUFA. These data highlight the importance of increase in the concentration of phospholipids and decrease in lyso-PL in maternal circulation so that intact phospholipids can be metabolized further by placental lipases for placental transfer. We have previously shown that the concentration of lyso-PL particularly those with PUFA is higher in cord blood compared to maternal plasma . The data presented here further strengthen the potential mechanism of maternal-foetal transfer of esterified fatty acids apart from several other lipid species. The increase in sphingomyelin and sphingosine-1-phosphate and decrease in ceramide-1-phosphate and total ceramide indicate the possible regulation of LCAT activity by sphingolipids. Sphingomyelin and sphingosine-1-phosphate have an inhibitory effect on LCAT whereas ceramide and ceramide-1-phosphate are its potent activators [40], and by orchestration of the differential concentration of sphingolipid subclasses, LCAT may be kept under tight regulation.

We observed nine patterns of the lipidomic changes from preconception to postpartum which revealed different responses of lipid species to the change in pregnancy status. These nine patterns can be summarized into four categories. First, the concentrations of around 80% of lipid species (pattern 2: decreased in pregnancy and pattern 4: increased in pregnancy) returned to the preconception concentrations after significant changes in pregnancy. Second, around 11% of lipid species (patterns 1, 3, 5 and 6) went through significant changes in pregnancy, but did not revert to the preconception concentrations at postpartum. These findings indicate the dysregulation of several plasma lipids due to pregnancy and may be associated with maternal metabolic outcomes in later life. These lipids were mainly from phospholipid (ether-link: 39 lipids and others: 10 lipids) and sphingolipid (18 lipids) classes. Third, around 7% of lipid species only showed significant changes at postpartum (patterns 7 and 8). Last, twelve lipid species (~ 2%) in pattern 9 (e.g. SM(44:3)) did not show any significant change across three time points. In addition, it is interesting to note that the ceramides containing long-chain fatty acids (pattern 4, 12 lipids, C16-C21) increased whereas very-long-chain (pattern 2, 19 lipids, C22-C26) fatty acids decreased in pregnancy. This finding suggests the differential regulation of ceramide synthases in pregnancy [41]. The widespread changes in lipid species and fine-tuning within individual lipid class highlight the extent of pregnancy-related changes in lipid metabolism not only for maternal-foetal transport but as well as for maternal metabolism.

We next investigated the association of plasma lipidomic profiles with cardiometabolic risk factors including body weight changes, pre-pregnancy adiposity and glycaemic traits. Although body weight change is a normal response to the physiological changes induced by pregnancy, excessive GWG and PWR may have adverse effects on both mother and child with immediate and long-lasting consequences [17, 42]. Breastfeeding has been reported to be associated with PWR [43], but this association was not observed in our study as the majority of mothers breastfed at 3 months postpartum. We observed that GWG was positively associated with PWR. The concentrations of ceramide, dehydrocholesterol, cholesteryl ester and ether-link phospholipids showed positive associations with GWG whereas these lipids were not significant with changes in WL. In the PWR analysis, opposing trends in several lipid species were observed with a noticeable change in the effect size of phospholipids and lysophospholipids compared to the GWG and WL analyses. The concentrations of several lipid species from phospholipid and lysophospholipid were negatively associated with PWR. Interestingly, we observed quite a few significant phospholipids in three association studies of body weight changes, but no overlapping phospholipids were found within the 43 common lipids. This observation highlights the differential regulation of phospholipid pathways in association with body weight changes. Our results highlight the importance of phospholipids and lysophospholipids in not only baseline metabolism but also the effects of pre-pregnancy obesity and excess weight retention on maternal metabolic health.

Pre-pregnancy adiposity has often been associated with pregnancy-related and postpartum outcomes [22]. Pre-pregnancy BMI has also been shown to influence the metabolic profiles in pregnancy, thus further highlighting the importance of preconceptional health [44, 45]. However, there are no studies on the longitudinal effect of ppBMI on lipidomic profiles before and after pregnancy. Our association studies showed the overarching effect of ppBMI on the lipidomic profiles not only in preconception but also in pregnancy and postpartum. We observed highly overlapped association results at two non-pregnant states (preconception and postpartum) and these BMI-related signatures were consistent with the findings in other adult populations [46]. There were three main differences between the association results of pregnant and non-pregnant states. Firstly, the association of ppBMI with lipidomic profiles showed divergent trends in phospholipids and sphingomyelins in two non-pregnant states whereas it exhibited a negative association in these lipid classes (excluding PC(18:0_20:3) and PC(18:0_20:4)) in pregnancy. As demonstrated in Fig. 1C, the concentrations of most of the phospholipids and sphingomyelins were increased from preconception to pregnancy as these are the major resources of polyunsaturated fatty acids and very long-chain fatty acids needed for foetal development. Our findings indicated that the mothers with higher ppBMI had lower concentrations of phospholipids and sphingomyelins in pregnancy (Fig. 4B). Secondly, most of diacylglycerol, alkyl-diacylglycerol and triacylglycerol lipids showed very significant positive association with ppBMI in two non-pregnant states, but only six lipids (common lipids in three analyses) were associated with ppBMI in pregnancy (Fig. 4B). Thirdly, amongst the pregnancy-related lipid signatures of ppBMI, a negative association was observed in several atypical lipid species including odd and branched-chain fatty acid containing phospholipid species (Fig. 4E). From these analyses, the common lipids at three time points could help understand the long-term effect of ppBMI on plasma lipidomic profiles (Fig. 4F). Ceramides with saturated fatty acids (particularly stearic acid) showed strong positive association whereas glycosphingolipids were negatively associated with ppBMI. The lipid species containing n-6 fatty acids (DGLA and ARA) showed consistent positive association with ppBMI. These findings provide evidence of the importance of ceramides, glycosphingolipids and n-6 fatty acids containing lipid species on maternal adiposity and cardiometabolic risk factors.

Pregnancy represents a unique physiological challenge that requires plasticity in systemic metabolism and the maintenance of glucose homeostasis is of particular importance [2]. Consistent with the previous reports [47, 48], we observed that FPG and HbA1c concentrations decreased in pregnancy and increased at postpartum whereas HOMA-IR, 2hPG and insulin concentrations showed the opposite trends. HbA1c, which reflects the average blood glucose concentration over the past 2 to 3 months [49], was associated with more lipid species than FPG and 2hPG. Our results also showed that circulating lipids are significantly more associated with fasting insulin and HOMA-IR than glucose concentrations (FPG, 2hPG and HbA1c) at three time points. Insulin promotes the synthesis of fatty acids in the liver and inhibits the breakdown of fat in adipose tissue [50, 51]. It has more profound association with lipid metabolism than FPG, 2hPG and HbA1c. HOMA-IR is derived from FPG and insulin concentrations, and we observed that it was associated with more lipid species than FPG and insulin separately. FPG showed the least significant association with lipidomic profiles in the pregnant state (Additional file 1: Fig. S8A). 2hPG showed very different association results with lipidomic profiles at three time points (Additional file 1: Fig. S8B) that may be due to the weak association between 2hPG at three time points. Amongst the 97 common lipid species in the HOMA-IR study, 87 of them showed positive association including diacylglycerol, alkyl-diacylglycerol and triacylglycerol lipid species containing FA16:0, FA18:0 and FA18:1, indicating increased de novo lipogenesis (DNL). DNL is not only associated with insulin resistance but is also involved in the pathogenesis of metabolic diseases including fatty liver with subsequent development of type 2 diabetes [52, 53]. The association studies of glycaemic traits with lipidomic profiles in pregnancy revealed significant changes in lysophospholipids in response to the fluctuation of glucose homeostasis in pregnancy. Recent studies have highlighted the role of lysophospholipids as signalling molecules to be involved in inflammation, insulin resistance and fatty liver disease that in turn are linked to obesity [54–56]. In this study, most of the lysophospholipids were negatively associated with ppBMI across the three physiological states except LPC 20:3. However, after adjusting for ppBMI, most of the lysophospholipids showed positive association with HOMA-IR in pregnancy. Studies have shown that IR is associated with differential concentrations of lysophospholipid [56, 57] and these can be subjected to dietary modulation to improve the metabolic outcomes [58]. However, as later stages of normal pregnancy are associated with IR, necessary to maintain the supply of nutrients for foetal accretion as well as for maternal metabolism, the overall positive association of lysophospholipids with HOMA-IR in pregnancy needs to be understood further.

Comparative assessment of measures of glucose homeostasis and insulin resistance in relation to the circulating lipids at preconception with a large sample size provided an integrated view of the effects of hyperglycaemia on systemic metabolism. We observed distinct lipidomic response to different measures, with the highest percentage of significant lipids at HOMA-IR, followed by insulin, 2hPG, HbA1c, IGT status and FPG. Overall, hyperglycaemia was associated with the accumulation of triacylglycerol, diacylglycerol, alkyl-diacylglycerol, ceramides, deoxy-ceramide, dihydroceramide and majority of phospholipids as well as several sphingomyelin and cholesteryl ester species. At the same time, glycosphingolipids, lysophospholipids and ether-linked phosphatidylcholines showed lower concentrations in hyperglycaemic conditions. The concentration of odd- or branched-chain containing phospholipids and very long-chain fatty acid (VLCFA) containing sphingolipids (i.e. SM(44:3) and Cer(d18:2/26:0)) as well as lysoalkylphosphatidylcholine (i.e. LPC(O-24:1) and LPC(O-24:2)) lipid species were lower in hyperglycaemic conditions. These branched-chain and very long-chain fatty acid are metabolized in peroxisomes and play an important role in carbohydrate metabolism [59]. Further, the odd-chain fatty acids had been reported to be inversely associated with type 2 diabetes [60]. Although most of the ceramides and sphingomyelins were positively associated with HOMA-IR, several species with atypical LCB (long chain base) d17:1 and dienic LCB d18:2 showed negative associations, in concordance with previous reports of the negative association of the latter with BMI and cardiovascular events in non-pregnant adult population [61, 62]. It was noteworthy that acylcarnitine species showed positive trends with 2hPG and negative trends with FPG, insulin, HOMA-IR and HbA1c in our study. Our findings highlight the accumulation of acylcarnitines was associated with IGT. The opposite trends of acylcarnitines with FPG and 2hPG were also observed in another adult population study [33, 63]. Although the differential levels of carnitine and acylcarnitine have been shown to be associated with GDM [64–66], the directionality of the associations needs further investigation. Lower concentrations in long-chain acylcarnitines have been shown to be associated with GDM [67]. However, studies have also shown a positive association between some of the long-chain and hydroxylated acylcarnitine with GDM [68]. In the current study, we only measured long-chain (non-hydroxylated) acylcarnitine that showed a consistent trend of negative association with glycaemic measures.

Early detection of GDM has attracted a lot of interest. The detailed alterations in lipid species for GDM have been studied in pregnancy [25, 69] including early pregnancy and samples across three trimesters; however, the inclusion of preconception lipidomic profiles offers novel insights into dysregulation of lipid metabolism for timely intervention. Our results revealed a panel of 37 lipid species with a potential to serve as a preconception molecular signature of GDM. Interestingly, 30 of them overlapped with the lipid species derived from comparative assessment of measures of glucose homeostasis and insulin resistance at preconception. These findings indicate that these 30 lipids were related to glycaemic traits at preconception and simultaneously associated with the GDM status diagnosed at 26–28 weeks of gestation. Compared to the controls, LPC(O-24:2) and SM(44:3) showed 9.88% and 10.52% lower concentration in GDM cases, respectively. Cer(d18:1/23:0), Cer(m18:0/20:0), Cer(m18:1/18:0), DG(16:0_16:0) and 24 triacylglycerol and alkyl-diacylglycerol lipids showed 9.92%, 23.97%, 19.98%, 13.67% and 11.50-34.13% higher concentration in GDM cases, respectively (Additional file 5: Table S4D). Amongst these, six triacylglycerol lipids (TG(48:0), TG(49:1), TG(50:1), TG(50:2), TG(51:1), TG(52:1)) were overlapped with the reported GDM markers at 10–14 weeks of gestation [25]. TG(51:1) was also reported as an early second-trimester predictive biomarker of GDM status [69]. In our study, TG(O-50:2) showed the most significant association (*P* = 7.66E−03 and 26.57% higher concentration in GDM cases) within triacylglycerol and alkyl-diacylglycerol lipids. These lipid species could serve as potential predictive lipid signatures of GDM at preconception and may help in the detection of GDM in early pregnancy. Overall, the concentration of several triacylglycerol, diacylglycerol, ceramides and deoxy-ceramides increased whereas lysophospholipids, phosphatidylinositol and sphingomyelins with diene long chain base decreased in GDM compared to non-GDM women. Several of these lipid species measured in early pregnancy were shown to be associated with the risk of GDM with the same directionality [25]. In addition, we observed ~89% of unique pregnancy-related lipid signatures of GDM status were from elevated glycerolipids (TG, TG(O) and DG) which indicated the important role of glycerolipids in pregnancy complications. The postpartum lipid signatures provided the evidence for the residual effect of GDM status on circulating lipids at 3 months postpartum.

Our study has some unique strengths. Longitudinal sampling and lipidomic analysis from preconception to postpartum period allowed us to identify the lipid signatures of metabolic adaptions and modifiable cardiometabolic risk factors. A large sample size at preconception enabled us to assess pre-pregnancy health status and may offer a critical window of opportunity for interventions with favourable outcomes. There are a few potential limitations of this study. First, the S-PRESTO cohort had a disproportionate number of Chinese participants over Malay, Indian or any combinations thereof, so ethnicity was adjusted in all the analyses. Second, as only less than half of the women recruited at preconception became pregnant, only a subset had available longitudinal lipidomics data at three time points. In addition, bias may exist in the self-reported variables (i.e. maternal education).

## Conclusions

We describe the longitudinal landscape of the circulating lipidome from preconception to postpartum in childbearing women. The differential changes in lipid species across different physiological states illustrate the complexity of metabolic adaptations for maternal-foetal transport and maternal metabolism. We identified lipid signatures linked with cardiometabolic risk factors including body weight change, pre-pregnancy obesity and glucose homeostasis and insulin resistance with potential implications both in pregnancy and postpartum life. These lipidomic signatures have potential to serve as biomarkers in addition to the standard lipid panel in assessment and prediction of cardiometabolic outcomes in women.

## Supplementary Information

**Additional file 1: Fig. S1.** Flowchart of sample selection and analysis steps in this study. **Fig. S2.** Pairwise Pearson correlation coefficient heat map of fasting glucose, 2-h post-load glucose, fasting insulin, HOMA-IR and HbA1c. **Fig. S3.** Histograms and pair-wise scatter plots of fasting glucose and 2-h post-load glucose concentrations at preconception, pregnancy and postpartum using trio subjects. **Fig. S4.** Histograms and pair-wise scatter plots of fasting insulin concentration and HOMA-IR at preconception, pregnancy and postpartum using trio subjects. **Fig. S5.** Histograms and pair-wise scatter plots of glycated haemoglobin (HbA1c, %) at preconception, pregnancy and postpartum using trio subjects. **Fig. S6.** Forest plots of fasting glucose concentration at postpartum, 2-h post-load glucose concentration at preconception, and HbA1c level at preconception, pregnancy and postpartum. **Fig. S7.** Scatter plots of effect sizes at preconception, pregnancy and postpartum in the fasting glucose, 2-h post-load glucose, fasting insulin, HOMA-IR association studies. **Fig. S8.** Venn diagrams of significant lipid species at preconception, pregnancy and postpartum for fasting glucose concentration, 2-h post-load glucose concentration and GDM status based on nominal p-value cut-off and the profiles of six selected lipid species from the 37 preconception signatures of GDM. **Fig. S9.** Association results of plasma fasting insulin concentration with plasma lipidomic profiles at preconception, pregnancy and postpartum. **Fig. S10.** Association results of fasting glucose, 2-h post-load glucose, impaired glucose tolerance status, fasting insulin, HOMA-IR and HbA1c levels at preconception. **Fig. S11.** Scatter plots of effect sizes in the association results of fasting glucose, 2-h post-load glucose, fasting insulin and HOMA-IR at preconception using trio and all subjects. **Fig. S12.** Percentage of individual lipid species within phosphatidylcholine, phosphatidylethanolamine and phosphatidylinositol classes. **Fig. S13.** Lipid ratios for enzyme indices of phosphatidylethanolamine n-methyltransferase (PEMT), lecithin-cholesterol acyltransferase (LCAT) and phospholipase A2 (PLA2).

**Additional file 2: Table S1. A.** Demographic, anthropometric and clinical characteristics of the study cohort. **B.** Comparison of characteristics between preconception, pregnancy and postpartum by paired t-test using trio subjects. **C.** Comparison of characteristics between pre-pregnant and non-pregnant subjects at preconception. **D.** The differences of lipidomic profiles between pre-pregnant and non-pregnant subjects at preconception. **E.** The group comparison results between preconception, pregnancy and postpartum using paired t-test and the patterns of lipid change.

**Additional file 3: Table S2. A.** The association results of plasma lipid changes against body weight changes. **B.** The association results of multivariate linear regression analysis for changes in lipid profile against body weight changes.

**Additional file 4: Table S3.** The association results of pre-pregnancy BMI with plasma lipid species at preconception, pregnancy and postpartum using trio subjects.

**Additional file 5: Table S4. A.** The association results of plasma fasting glucose concentration with plasma lipid species at preconception, pregnancy and postpartum using trio subjects. **B.** The association results of plasma 2h post-load glucose concentration with plasma lipid species at preconception, pregnancy and postpartum using trio subjects. **C.** The association results of GDM status with plasma lipid species at preconception, pregnancy and postpartum using trio subjects. **D.** A panel of 37 lipid species as a potential preconception molecular signature of gestational diabetes mellitus. **E.** The association results of plasma fasting insulin concentration with plasma lipid species at preconception, pregnancy and postpartum using trio subjects. **F.** The association results of HOMA-IR level with plasma lipid species at preconception, pregnancy and postpartum using trio subjects. **G.** The association results of HbA1c (%) with plasma lipid species at preconception, pregnancy and postpartum using trio subjects.

**Additional file 6: Table S5.** The association results of glycaemic traits with plasma lipid species at preconception using 936 subjects.

**Additional file 7: Table S6.** The 87 lipid signatures of pre-pregnant vs. non-pregnant analysis and their corresponding results for ppBMI and glycaemic traits at preconception.

## Data Availability

The data supporting the findings and figures in this study are provided in the supplementary materials. Other data are not publicly available due to ethical restrictions but can be obtained from the authors upon reasonable request and subject to appropriate approvals, including from the S-PRESTO cohort’s Executive Committee.

## Abbreviations

AC: Acylcarnitine
ARA: Arachidonic acid
BMI: Body mass index
CE: Cholesteryl ester
Cer: Ceramide
Cer1P: Ceramide-1-phosphate
COH: Free cholesterol
DE: Dehydrocholesterol
deoxyCer: Deoxy-ceramide
DG: Diacylglycerol
DHA: Docosahexaenoic acid
dhCer: Dihydroceramide
GM3: GM3 ganglioside
GWG: Gestational weight gain
HbA1c: Glycated haemoglobin
HDL: High-density lipoprotein
Hex2Cer: Dihexosylceramide
Hex3Cer: Trihexosylceramide
HexCer: Monohexosylceramide
HOMA-IR: Homeostatic model assessment for insulin resistance
LC-MS/MS: Liquid chromatography mass spectrometry
LDL: Low-density lipoprotein
LPC: Lysophosphatidylcholine
LPC(O): Lysoalkylphosphatidylcholine
LPC(P): Lysoalkenylphosphatidylcholine
LPE: Lysophosphatidylethanolamine
LPE(P): Lysoalkenylphosphatidylethanolamine
LPI: Lysophosphatidylinositol
LPS: Lysophosphatidylserine
NL: Neutral loss
PC: Phosphatidylcholine
PC(O): Alkylphosphatidylcholine
PC(P): Alkenylphosphatidylcholine
PE: Phosphatidylethanolamine
PE(O): Alkylphosphatidylethanolamine
PE(P): Alkenylphosphatidylethanolamine
PG: Phosphatidylglycerol
PI: Phosphatidylinositol
PS: Phosphatidylserine
PWR: Postpartum weight retention
S1P: Sphingosine-1-phosphate
SM: Sphingomyelin
Sph: Sphingosine
TG: Triacylglycerol
TG(O): Alkyl-diacylglycerol
WL: Weight loss
