# Supplementary material for: Plasma lipidomic profiling reveals metabolic adaptations to pregnancy and signatures of cardiometabolic risk: a preconception and longitudinal cohort study
Source: BMC Med. 2023 Feb 13;21:53. doi: 10.1186/s12916-023-02740-x (PMC9926745; doi:10.1186/s12916-023-02740-x)
Supplement: Supplementary file 1 — Additional file 1: Fig. S1. Flowchart of sample selection and analysis steps in this study. Fig. S2. Pairwise Pearson correlation coefficient heat map of fasting glucose, 2-h post-load glucose, fasting insulin, HOMA-IR and HbA1c. Fig. S3. Histograms and pair-wise scatter plots of fasting glucose and 2-h post-load glucose concentrations at preconception, pregnancy and postpartum using trio subjects. Fig. S4. Histograms and pair-wise scatter plots of fasting insulin concentration and HOMA-IR at preconception, pregnancy and postpartum using trio subjects. Fig. S5. Histograms and pair-wise scatter plots of glycated haemoglobin (HbA1c, %) at preconception, pregnancy and postpartum using trio subjects. Fig. S6. Forest plots of fasting glucose concentration at postpartum, 2-h post-load glucose concentration at preconception, and HbA1c level at preconception, pregnancy and postpartum. Fig. S7. Scatter plots of effect sizes at preconception, pregnancy and postpartum in the fasting glucose, 2-h post-load glucose, fasting insulin, HOMA-IR association studies. Fig. S8. Venn diagrams of significant lipid species at preconception, pregnancy and postpartum for fasting glucose concentration, 2-h post-load glucose concentration and GDM status based on nominal p-value cut-off and the profiles of six selected lipid species from the 37 preconception signatures of GDM. Fig. S9. Association results of plasma fasting insulin concentration with plasma lipidomic profiles at preconception, pregnancy and postpartum. Fig. S10. Association results of fasting glucose, 2-h post-load glucose, impaired glucose tolerance status, fasting insulin, HOMA-IR and HbA1c levels at preconception. Fig. S11. Scatter plots of effect sizes in the association results of fasting glucose, 2-h post-load glucose, fasting insulin and HOMA-IR at preconception using trio and all subjects. Fig. S12. Percentage of individual lipid species within phosphatidylcholine, phosphatidylethanolamine and phosphatidylinositol classe [file 12916_2023_2740_MOESM1_ESM.docx]

Supplementary Materials for

**Plasma lipidomic profiling reveals metabolic adaptations to pregnancy and signatures of cardiometabolic risk: a preconception and longitudinal cohort study**

Li Chen, Sartaj Ahmad Mir, Anne K. Bendt, Chua W.L. Esther, Kothandaraman Narasimhan, Karen Mei-Ling Tan, See Ling Loy, Kok Hian Tan, Lynette Shek, Jerry Chan, Fabian Yap, Michael J. Meaney, Shiao-Yng Chan, Yap Seng Chong, Peter D. Gluckman, Johan G. Eriksson, Neerja Karnani, Markus R. Wenk

**Additional File 1: Fig. S1-S13**

**Fig. S1** Flowchart of sample selection and analysis steps in this study

**Fig. S2** Pairwise Pearson correlation coefficient heat map of fasting glucose, 2-h post-load glucose, fasting insulin, HOMA-IR and HbA1c

**Fig. S3** Histograms and pair-wise scatter plots of fasting glucose and 2-h post-load glucose concentrations at preconception, pregnancy and postpartum using trio subjects

**Fig. S4** Histograms and pair-wise scatter plots of fasting insulin concentration and HOMA-IR at preconception, pregnancy and postpartum using trio subjects

**Fig. S5** Histograms and pair-wise scatter plots of glycated haemoglobin (HbA1c, %) at preconception, pregnancy and postpartum using trio subjects

**Fig. S6** Forest plots of fasting glucose concentration at postpartum, 2-h post-load glucose concentration at preconception, and HbA1c level at preconception, pregnancy and postpartum

**Fig. S7** Scatter plots of effect sizes at preconception, pregnancy and postpartum in the fasting glucose, 2-h post-load glucose, fasting insulin, HOMA-IR association studies

**Fig. S8** Venn diagrams of significant lipid species at preconception, pregnancy and postpartum for fasting glucose concentration, 2-h post-load glucose concentration and GDM status based on nominal p-value cut-off and the profiles of six selected lipid species from the 37 preconception signatures of GDM

**Fig. S9** Association results of plasma fasting insulin concentration with plasma lipidomic profiles at preconception, pregnancy and postpartum

**Fig. S10** Association results of fasting glucose, 2-h post-load glucose, impaired glucose tolerance status, fasting insulin, HOMA-IR and HbA1c levels at preconception

**Fig. S11** Scatter plots of effect sizes in the association results of fasting glucose, 2-h post-load glucose, fasting insulin and HOMA-IR at preconception using trio and all subjects.

**Fig. S12** Percentage of individual lipid species within phosphatidylcholine, phosphatidylethanolamine and phosphatidylinositol classes

**Fig. S13** Lipid ratios for enzyme indices of phosphatidylethanolamine n-methyltransferase (PEMT), lecithin-cholesterol acyltransferase (LCAT) and phospholipase A2 (PLA2).

**S-PRESTO Cohort**

**(N=1032)**

Based on availability and quality of plasma samples

**Plasma lipidomics at three time points**

**preconception (N=976), pregnancy (N=337) and postpartum (N=282)**

**Adaptations in lipid metabolism to pregnancy**

**Step 1: Lipidomic differences between pre-pregnant (n=360) and non-pregnant (n=494) subjects at preconception**

**Lipidomic profiles of trio subjects (n=263)**

**Step 2: Longitudinal changes in lipid concentration (paired t-test)**

**Step 3: Patterns of lipid changes across three time points**

**Lipid signatures of cardiometabolic risk traits**

**Step 4: Association of changes in lipid concentration with body weight changes**

**Step 5: Association of pre-pregnancy BMI with lipidomic profiles at three time points**

**Step 6: Association of measures of glucose homeostasis and insulin resistance with lipidomic profiles at three time points**

**Step 7: Association of measures of glucose homeostasis and insulin resistance with lipidomic profiles at preconception (n=936)**

**Fig. S1.** Flowchart of sample selection and analysis steps in this study

**Postpartum**

**Pregnancy**

**Preconception**

**Postpartum**

**Pregnancy**

**Preconception**

**Fig. S2.** Pairwise Pearson correlation coefficient heat map (R) of fasting glucose (FPG, mmol/L), 2-h post-load glucose (2hPG, mmol/L), fasting insulin (mU/mL), HOMA-IR and HbA1c (%) at preconception, pregnancy and postpartum.

**A**  **Fasting Glucose Concentration (mmol/L)**


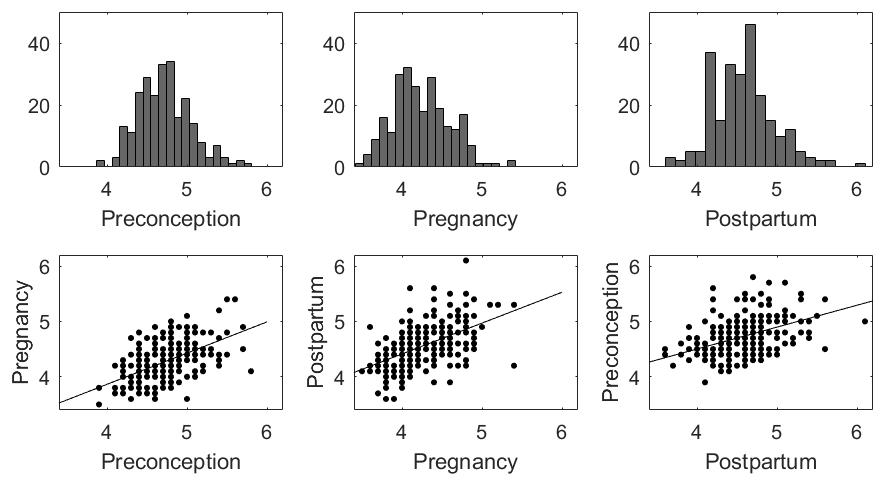


Count

4.56±0.38

4.73±0.34

4.27±0.35

R^2^=0.27

R^2^=0.29

R^2^=0.20

**B**  **2-h post-load glucose concentration (mmol/L)**


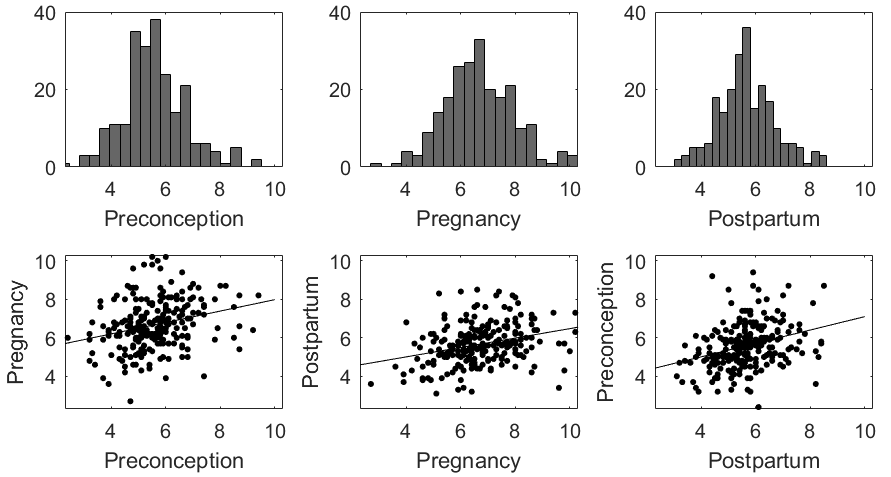


Count

6.67±1.29

5.66±1.05

5.59±1.17

R^2^=0.10

R^2^=0.09

R^2^=0.07

**Fig. S3** Histograms and pair-wise scatter plots of (**A**) fasting glucose concentration (n=249, mmol/L) and (**B**) 2-h post-load glucose concentration (n=226, mmol/L) at preconception, pregnancy and postpartum using trio subjects

**A Fasting Insulin Concentration (mU/mL)**


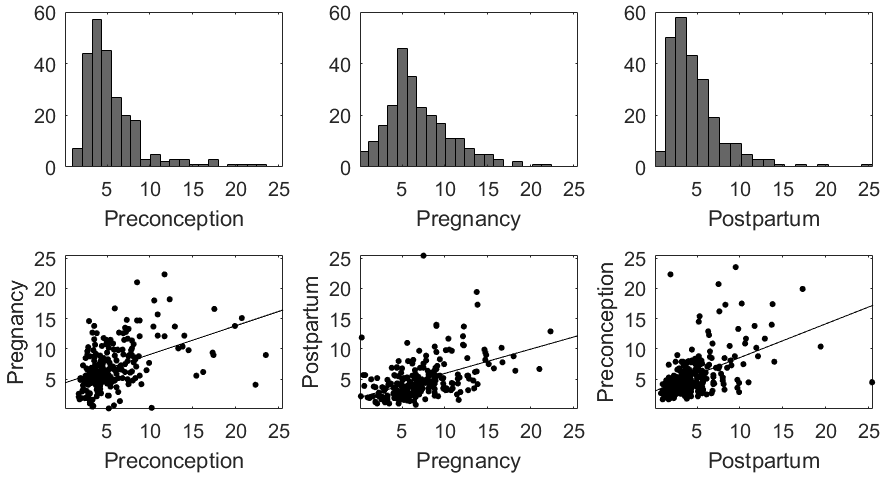


Count

4.80±3.20

7.07±3.78

5.70±3.61

R^2^=0.22

R^2^=0.24

R^2^=0.20

**B HOMA-IR**


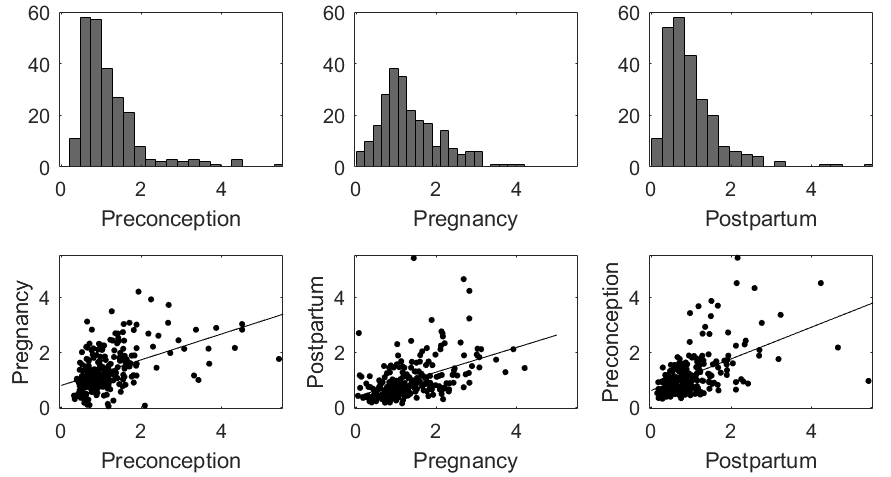


Count

1.00±0.72

1.35±0.76

1.19±0.78

R^2^=0.28

R^2^=0.23

R^2^=0.22

**Fig. S4** Histograms and pair-wise scatter plots of (**A**) fasting insulin concentration (n=243, mU/mL) and (**B**) HOMA-IR (n=240) at preconception, pregnancy and postpartum using trio subjects

**HbA1c (%)**


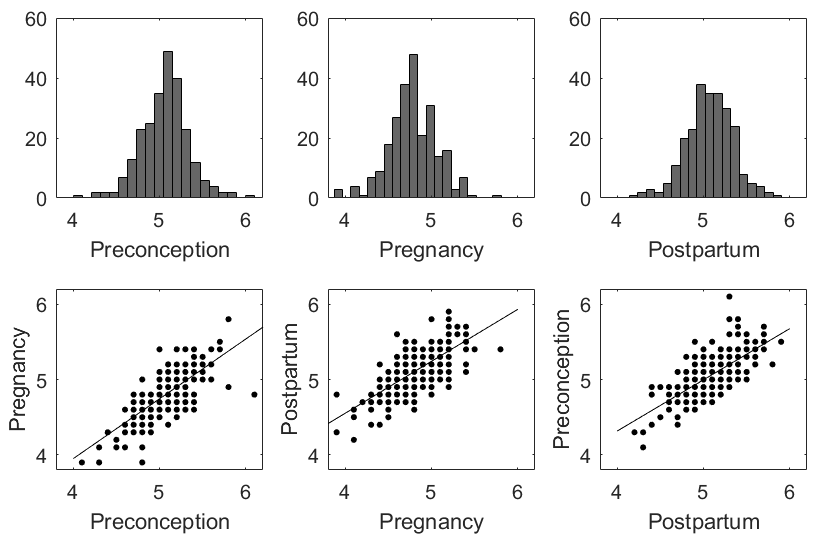


R^2^=0.51

R^2^=0.54

R^2^=0.51

5.06±0.27

4.79±0.29

5.10±0.28

Count

**Fig. S5** Histograms and pair-wise scatter plots of glycated haemoglobin (HbA1c, %) at preconception, pregnancy and postpartum using trio subjects (n=249)

**A B**


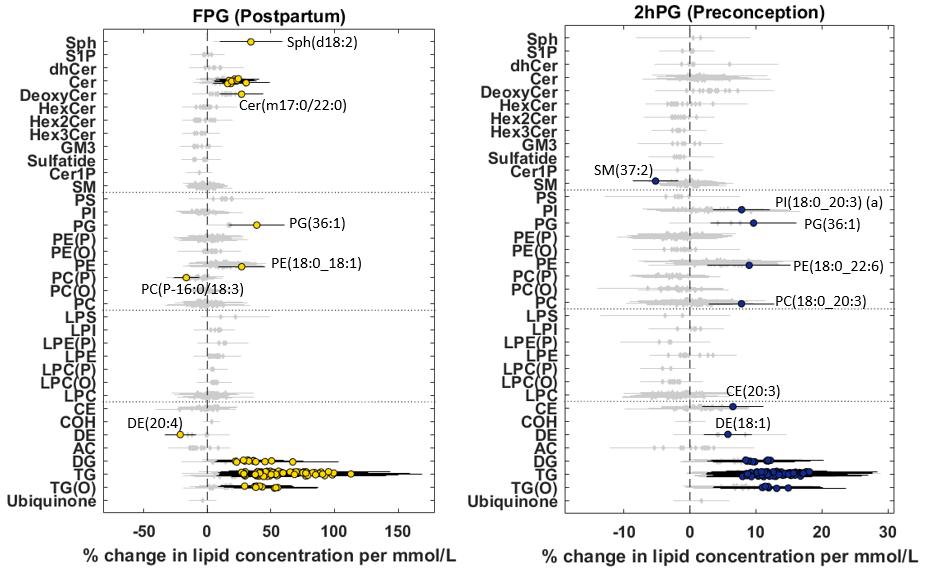


**C**


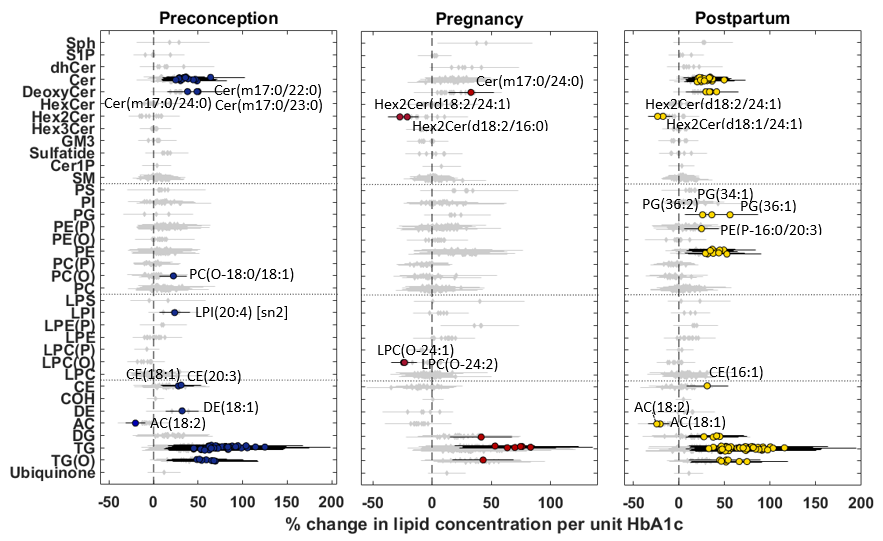


**Fig. S6** Forest plots of the association results of (**A**) fasting glucose concentration (FPG) at postpartum, (**B**) 2-h post-load glucose concentration (2hPG) at preconception, and (**C**) HbA1c level (%) at preconception, pregnancy and postpartum. Diamond – P_adj_ ≥ 0.05 (grey) and circle – P_adj_ <0.05 in forest plots. Error bar – 95% confidence interval.


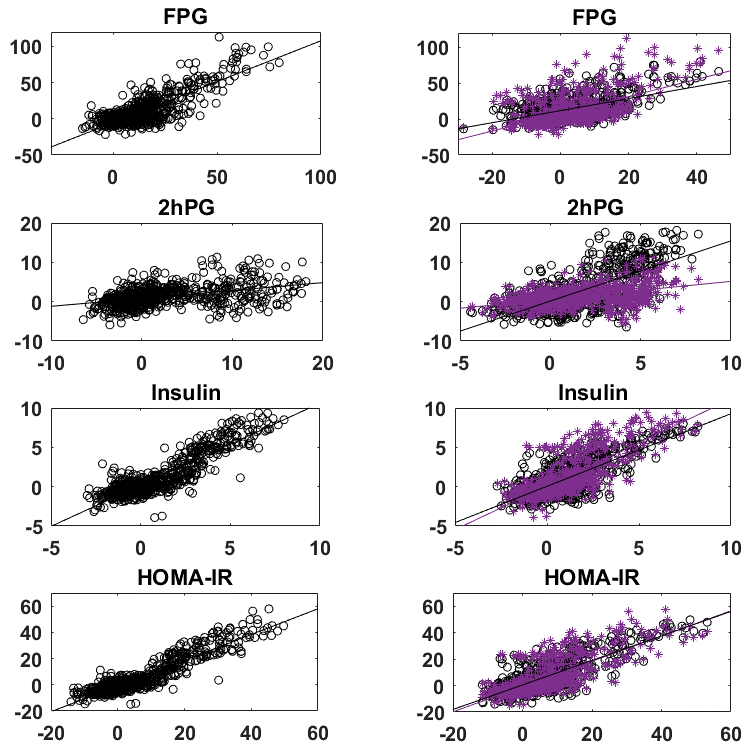
 **A**  **B**

R^2^=0.30

R^2^=0.65

R^2^=0.32

R^2^=0.18

R^2^=0.47

R^2^=0.18

**Effect Size (Postpartum)**

R^2^=0.80

R^2^=0.57

**Effect Size (o-Preconception; *-Postpartum)**

R^2^=0.60

R^2^=0.85

R^2^=0.63

R^2^=0.59


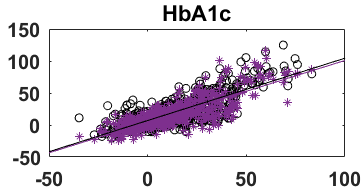

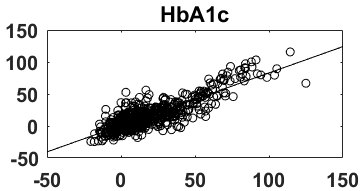


R^2^=0.64

R^2^=0.73

R^2^=0.67

**Effect Size (Pregnancy)**

**Effect Size (Preconception)**

**Fig. S7** Scatter plots of effect sizes at preconception, pregnancy and postpartum in the association studies of fasting glucose concentration (FPG, mmol/L), 2-h post-load glucose concentration (2hPG, mmol/L), fasting insulin concentration (mU/mL), HOMA-IR and HbA1c level (%). (**A**) Postpartum vs. Preconception, (**B**) Preconception or Postpartum vs. Pregnancy. Effect size is % change in lipid concentration per mmol/L (FPG and 2hPG) or per mU/mL (Insulin) or per unit HOMA-IR or per unit HbA1c.


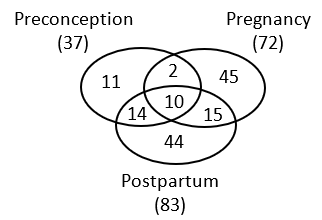

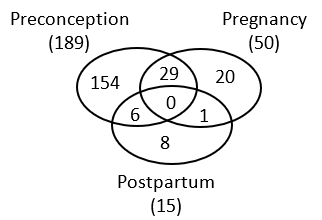


**GDM**

**C**

**B**

**A**


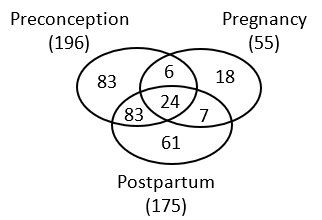


**2hPG**

**FPG**

**D**


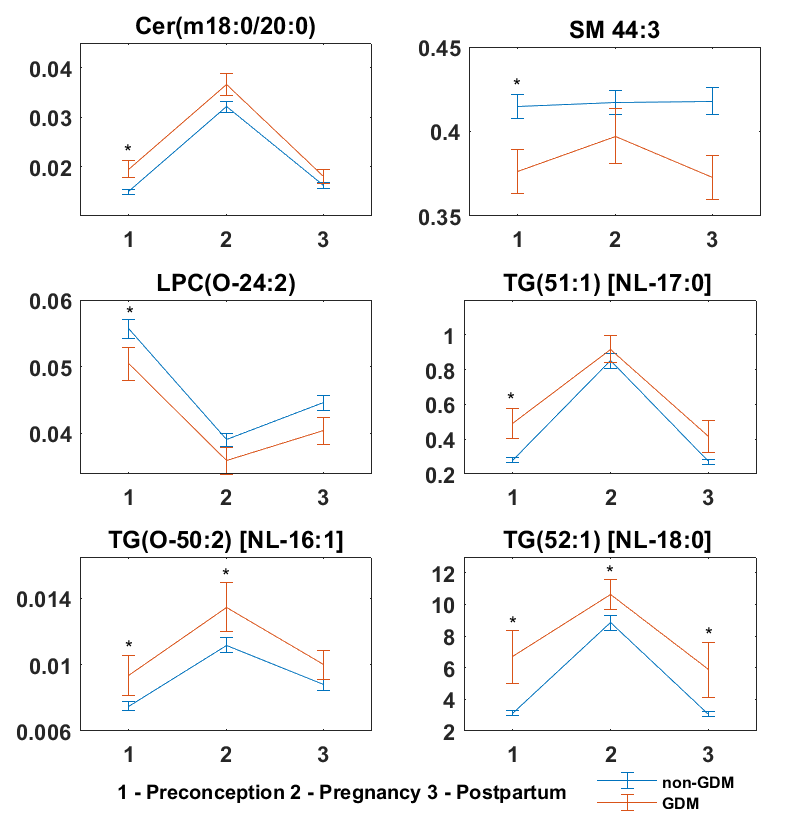


**Lipid Concentration (µmol/L)**

**Fig. S8** Venn diagrams of significant lipid species at preconception, pregnancy and postpartum for (**A**) fasting glucose concentration, (**B**) 2-h post-load glucose concentration, (**C**) GDM status based on nominal p-value cut-off (<0.05) and (**D**) the profiles of six selected lipid species from the 37 preconception signatures of GDM (* - nominal p-value < 0.05). Related to **Table S4A-D**.

**Fig. S9** Association results of plasma fasting insulin concentration with plasma lipidomic profiles at preconception, pregnancy and postpartum using trio subjects. (**A**) Forest plots, and (**B**) Scatter plots of effect sizes in the fasting insulin (% change in lipid concentration per mU/mL insulin) and HOMA-IR association studies (% change in lipid concentration per unit HOMA-IR)


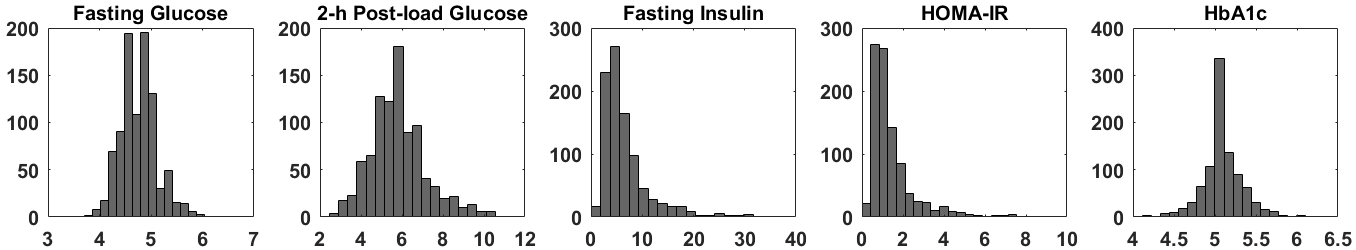
**A**

Count

**HOMA-IR**

**HbA1c (%)**

**Insulin (mU/mL)**

**2hPG (mmol/L)**

**FPG (mmol/L)**

**B**

**C**


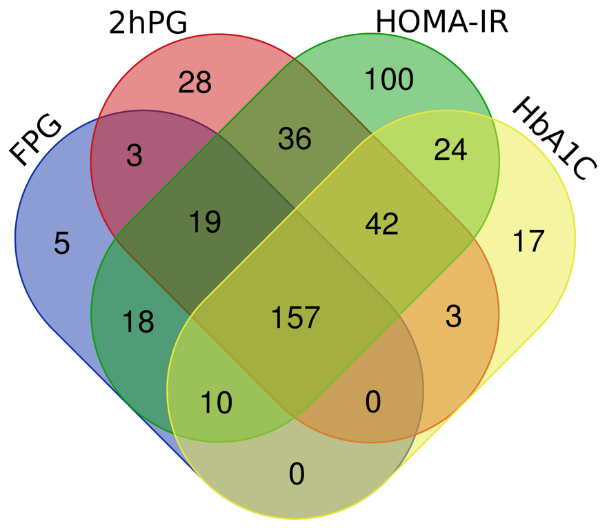


**D**

**Fig. S10** Association results of fasting glucose concentration (FPG), 2-h post-load glucose concentration (2hPG), impaired glucose tolerance status (IGT vs. Normal), fasting insulin concentration, HOMA-IR and HbA1c (%) at preconception (n=936). (**A**) Histograms, (**B**) Pairwise Pearson correlation heat map (R^2^) of six variables, (**C**) Pairwise Pearson correlation heat map (R^2^) of effect sizes in the association studies of six variables with plasma lipidomic profiles, and (**D**) Venn-diagram of significant lipids in the FPG, 2hPG, HOMA-IR and HbA1c studies.


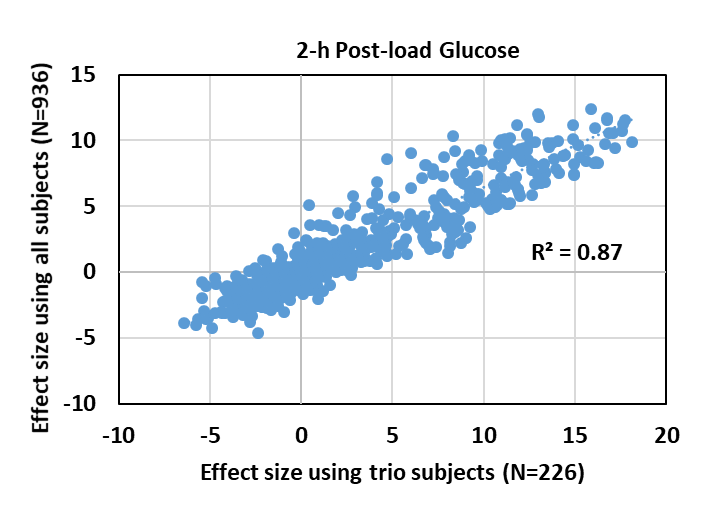

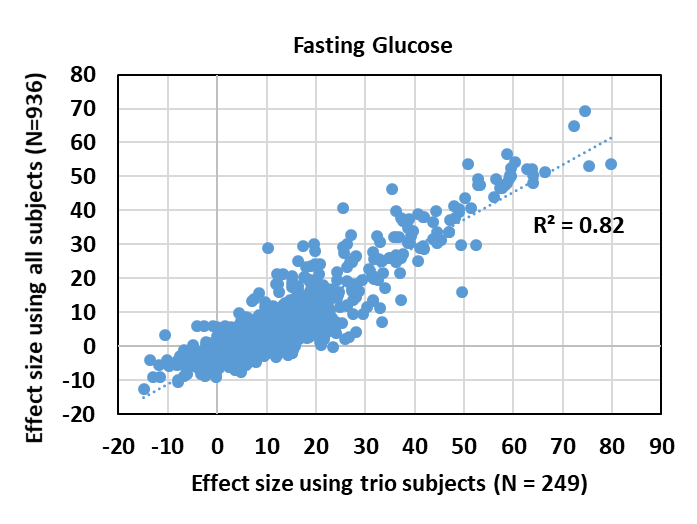

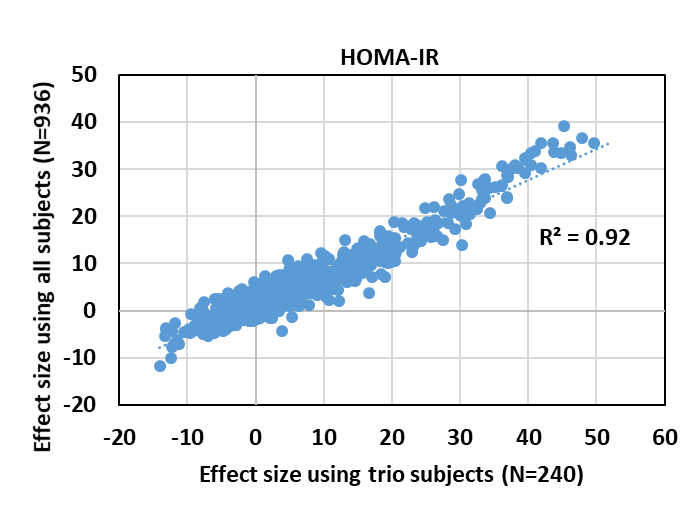

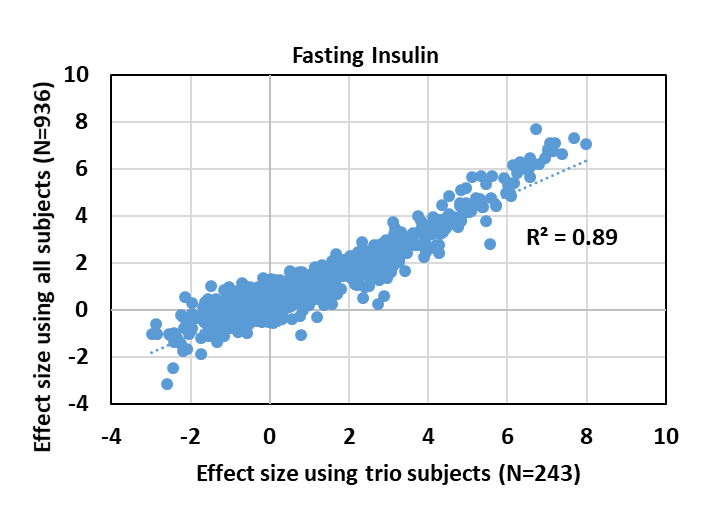

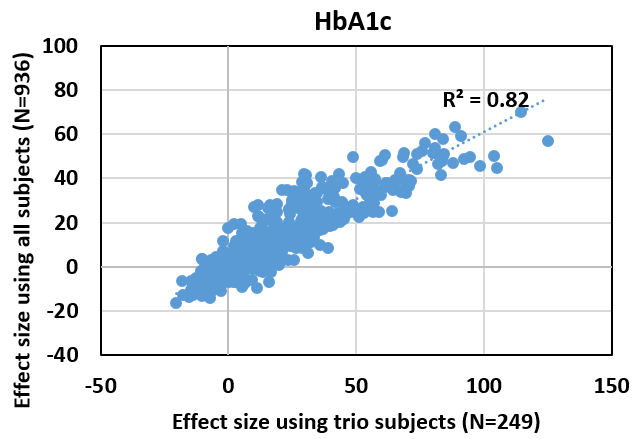


**Fig. S11** Scatter plots of effect sizes in the association results of fasting glucose (FPG), 2-h post-load glucose (2hPG), fasting insulin, HOMA-IR and HbA1c at preconception using trio and all subjects. Effect sizes are presented by % change in lipid concentration per mmol/L in FPG and 2hPG, per mU/mL insulin, per unit HOMA-IR and per unit HbA1c (%).

**A**


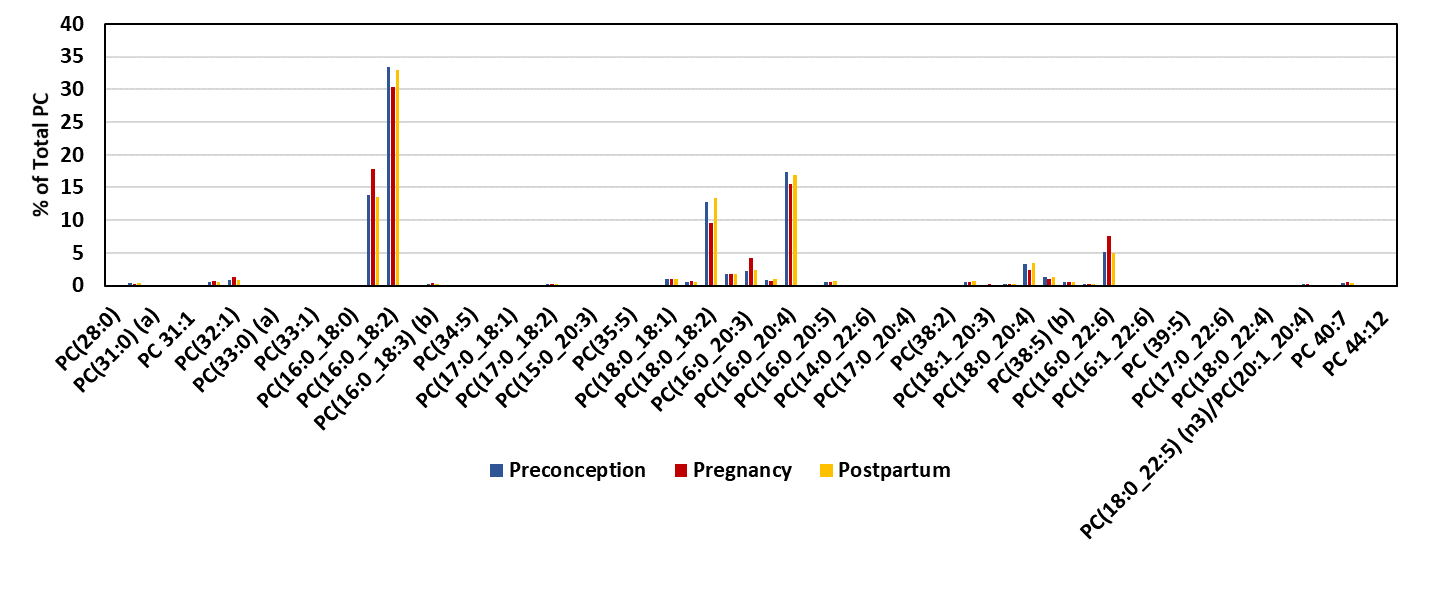


**B**


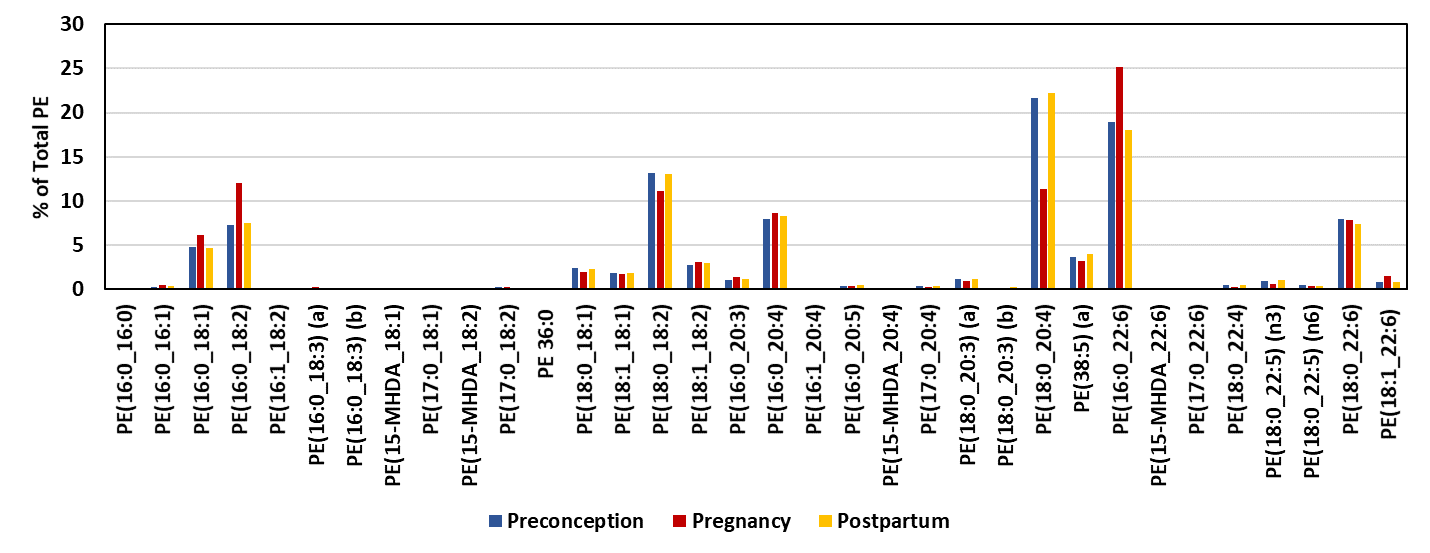


**C**


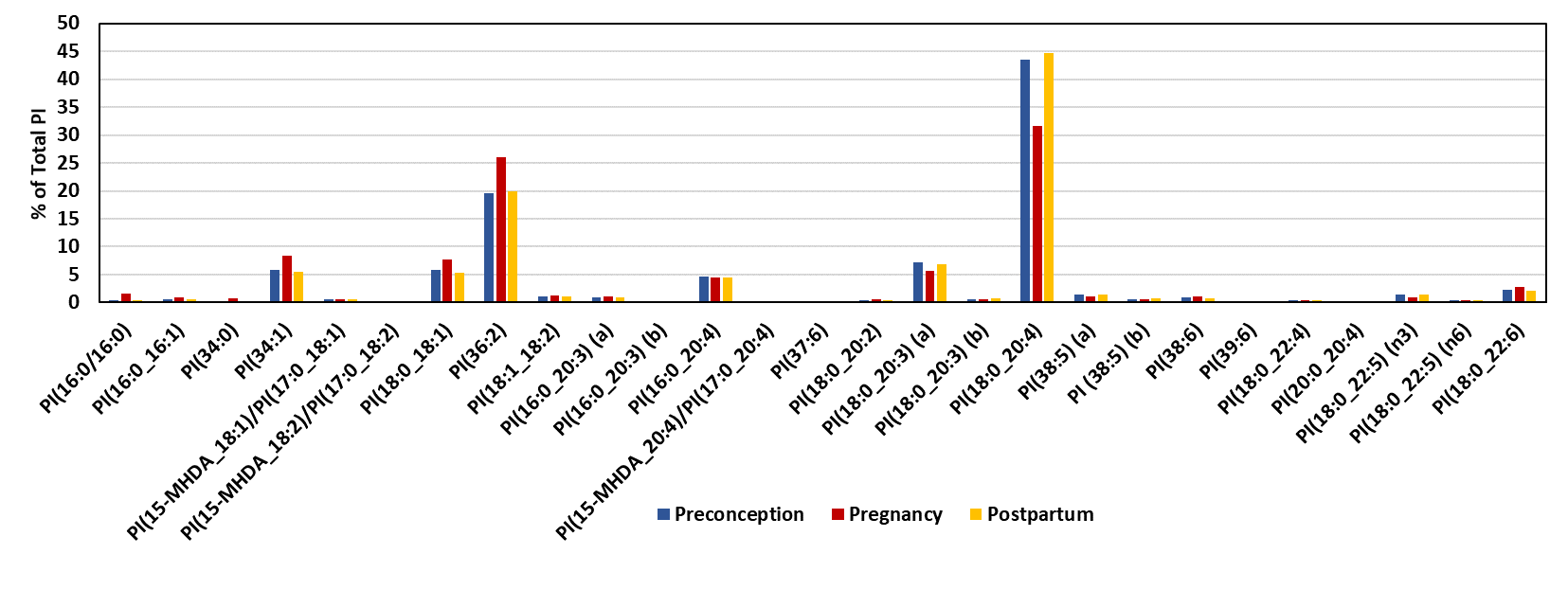


**Fig. S12** Percentage of each lipid species within (**A**) phosphatidylcholine (PC), (**B**) phosphatidylethanolamine (PE), and (**C**) phosphatidylinositol (PI) classes.


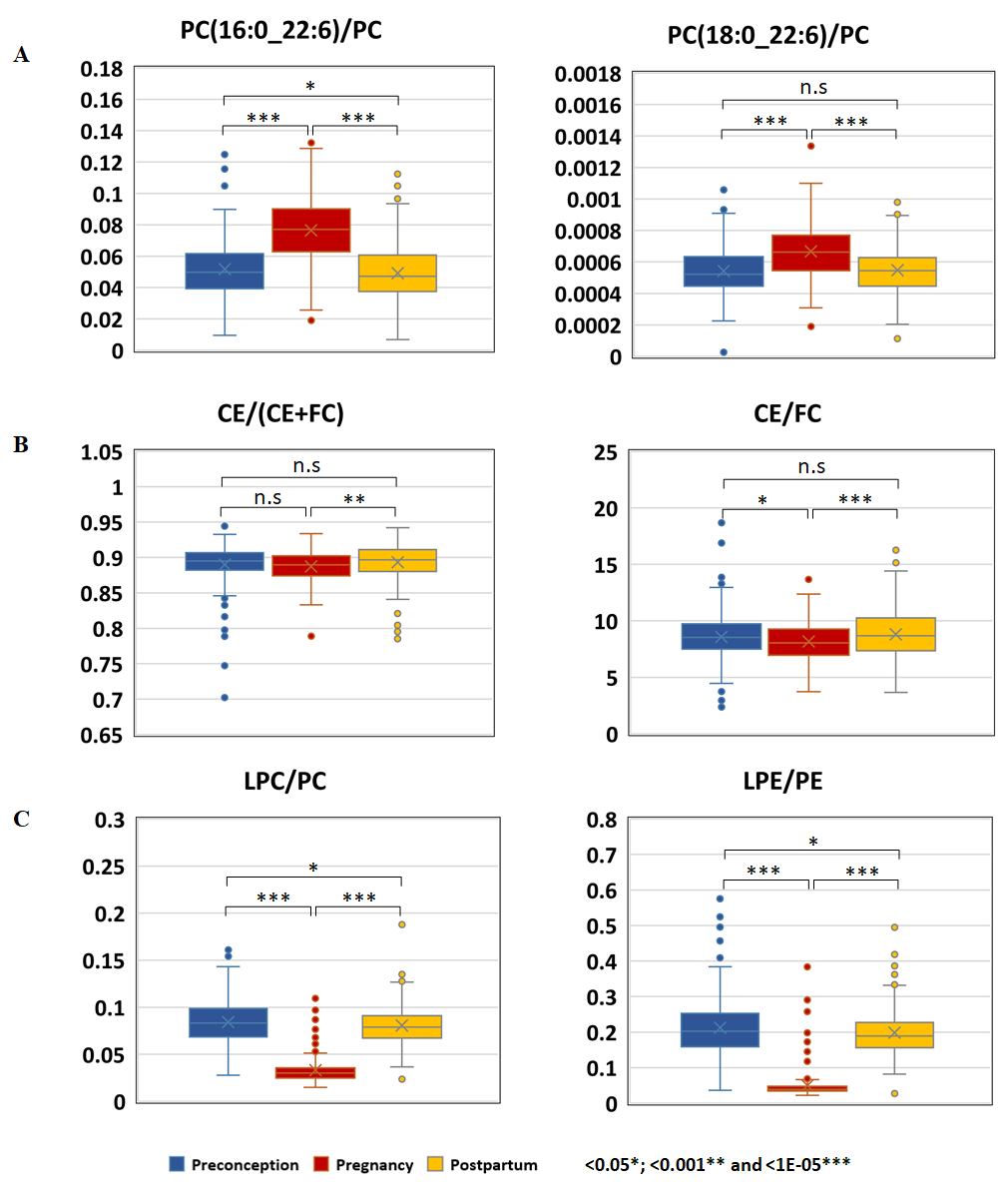


**Ratio**

**Ratio**

**Ratio**

**Fig. S13** Lipid ratios for enzyme indices of phosphatidylethanolamine n-methyltransferase (PEMT), lecithin-cholesterol acyltransferase (LCAT) and phospholipase A2 (PLA2). (**A**) PEMT, (**B**) LCAT, and (**C**) PLA2
